# Recombinant ACE2 protein protects against acute lung injury induced by SARS-CoV-2 spike RBD protein

**DOI:** 10.1186/s13054-022-04034-9

**Published:** 2022-06-09

**Authors:** Lingbing Zhang, Yandan Zhang, Xia Qin, Xuejun Jiang, Jun Zhang, Lejiao Mao, Ziqi Jiang, Yu Jiang, Gang Liu, Jingfu Qiu, Chengzhi Chen, Feng Qiu, Zhen Zou

**Affiliations:** 1 Department of Pharmacy, The First Affiliated Hospital of Chongqing Medical University, Chongqing, 400016 People’s Republic of China; 2 Molecular Biology Laboratory of Respiratory Disease, Institute of Life Sciences, Chongqing Medical University, Chongqing, 400016 People’s Republic of China; 3 Center of Experimental Teaching for Public Health, Experimental Teaching and Management Center, Chongqing Medical University, Chongqing, 400016 People’s Republic of China; 4 Research Center for Environment and Human Health, School of Public Health, Chongqing Medical University, Chongqing, 400016 People’s Republic of China; 5 Department of Respiratory Medicine, The University-Town Hospital of Chongqing Medical University, Chongqing, 401331 People’s Republic of China; 6 Department of Emergency, The University-Town Hospital of Chongqing Medical University, Chongqing, 401331 People’s Republic of China; 7 Department of Health Laboratory Technology, School of Public Health, Chongqing Medical University, Chongqing, 400016 People’s Republic of China; 8 Department of Occupational and Environmental Health, School of Public Health, Chongqing Medical University, Chongqing, 400016 People’s Republic of China

**Keywords:** SARS-CoV-2, Spike RBD, ACE2, Angiotensin II, Acute lung injury

## Abstract

**Background:**

SARS-CoV-2 infection leads to acute lung injury (ALI) and acute respiratory distress syndrome (ARDS). Both clinical data and animal experiments suggest that the renin–angiotensin system (RAS) is involved in the pathogenesis of SARS-CoV-2-induced ALI. Angiotensin-converting enzyme 2 (ACE2) is the functional receptor for SARS-CoV-2 and a crucial negative regulator of RAS. Recombinant ACE2 protein (rACE2) has been demonstrated to play protective role against SARS-CoV and avian influenza-induced ALI, and more relevant, rACE2 inhibits SARS-CoV-2 proliferation in vitro. However, whether rACE2 protects against SARS-CoV-2-induced ALI in animal models and the underlying mechanisms have yet to be elucidated.

**Methods and Results:**

Here, we demonstrated that the SARS-CoV-2 spike receptor-binding domain (RBD) protein aggravated lipopolysaccharide (LPS)-induced ALI in mice. SARS-CoV-2 spike RBD protein directly binds and downregulated ACE2, leading to an elevation in angiotensin (Ang) II. AngII further increased the NOX1/2 through AT_1_R, subsequently causing oxidative stress and uncontrolled inflammation and eventually resulting in ALI/ARDS. Importantly, rACE2 remarkably reversed SARS-CoV-2 spike RBD protein-induced ALI by directly binding SARS-CoV-2 spike RBD protein, cleaving AngI or cleaving AngII.

**Conclusion:**

This study is the first to prove that rACE2 plays a protective role against SARS-CoV-2 spike RBD protein-aggravated LPS-induced ALI in an animal model and illustrate the mechanism by which the ACE2-AngII-AT_1_R-NOX1/2 axis might contribute to SARS-CoV-2-induced ALI.

## Background

Angiotensin-converting enzyme 2 (ACE2) is ubiquitously expressed in multiple organs, including the lung, heart, small intestine and kidney [1, 2]. Similar to SARS-CoV, the functional/major receptor for SARS-CoV-2 has also been proven to be ACE2 [3, 4]. SARS-CoV-2 has a surface-anchored spike glycoprotein with surface receptor binding domains (RBDs). The entry of SARS-CoV-2 is mediated by the spike RBD, which binds the ACE2 receptor and is then primed by transmembrane protease serine 2 (TMPRSS2). Subsequently, the fusion of viral and cellular membranes and the entry of SARS-CoV-2 are completed [5, 6]. Therefore, interrupting the interaction between the spike RBD and ACE2 serves as a potent target for the development of a remedy strategy for SARS-CoV-2 infection [7].

Functionally, in addition serving as a receptor for SARS-CoV-2, ACE2 is a negative regulator of the renin–angiotensin system (RAS). The RAS is a hormone system that mainly modulates blood pressure, maintains systemic vascular resistance and balances fluid and electrolytes [8, 9]. In the plasma, angiotensin (Ang) I is generated by the cleavage of angiotensinogen by renin. AngI is physiologically inactive until it is cleaved to generate bioactive angiotensin II (AngII) by angiotensin-converting enzyme (ACE). ACE is a homologue of ACE2, which primarily exists in the vascular endothelium of the lungs and kidneys [10]. AngII is the main effector molecule of RAS, contributing to an elevation in blood pressure through angiotensin II type 1 receptor (AT_1_R), or to a lesser extent, AT_2_R [11]. In contrast to the function of ACE, ACE2 catalyzes AngII to generate Ang1-7, which selectively activates the Mas receptor. Ang1-7/Mas axis reversely modulates various AngII actions. In addition, ACE2 may catalyze AngI to generate Ang1-9, which also helps diminish the effect of AngII [12]. Thus, ACE2/Ang1-7/Mas receptor axis is known to negatively regulate the excessively activated ACE/AngII/AT_1_R axis in cardiovascular diseases [13, 14]. In particular, clinical data from COVID-19 patients strongly suggest that the RAS system is closely linked to the severity and outcome of COVID-19, although a conclusion regarding the role of AngII in SARS-CoV-2 infection remains controversial [15]. Most studies support the notion that the elevation in AngII triggered by the interaction between ACE2 and SARS-CoV-2 spike RBD protein may be an important pathogenic factor in critically ill COVID-19 patients. Liu et al. reported that the AngII level in plasma samples from SARS-CoV-2 virus-infected patients (12 cases) was markedly elevated and linearly associated with the viral load and lung injury, suggesting the critical role of elevated AngII in SARS-CoV-2 virus infection-induced lung injury [16]. Wu et al. investigated 82 COVID-19 patients and found that the plasma AngII levels in critically ill COVID-19 patients were significantly higher than those in the controls and those with mild COVID-19 symptoms. These authors revealed a positive correlation between the plasma AngII levels and COVID-19 severity [17]. Similarly, Osman et al. found higher concentrations of AngII and AngI in the plasma of 44 COVID-19 patients; however, the plasma levels of Ang1-7 were stable in prolonged viral shedders [18]. Altogether, these clinical observations highlight the possible link between elevated AngII levels and the development and outcome of SARS-CoV-2 infection. In contrast, a few studies indicated increased soluble ACE2 levels and decreased AngII levels in critically ill COVID-19 patients (*n* = 10) compared to healthy controls. This discrepancy might be due to the very small cohort included [19]. Dynamic observations of the changes in AngII in a larger cohort COVID-19 patients could be beneficial for confirming the role of AngII during SARS-CoV-2 induced ALI/ARDS.

Considering that rACE2 has been reported to reverse ALI induced by SARS-CoV [20, 21], H5N1 avian influenza virus [22] and H7N9 avian influenza virus [23], rACE2 likely has the potential to treat ALI induced by SARS-CoV-2. More strikingly, recombinant ACE2 protein (rACE2) exerts a positive role in one COVID-19 patient [24], suggesting the benefits of modulating RAS by supplementation with ACE2 [25]. However, the role and underlying mechanism of rACE2 in SARS-CoV-2 infection are largely unknown, which may be partially due to the deficiency of an appropriate animal model. Because of phylogenetic differences in ACE2, conventional mice are resistant to SARS-CoV-2 infection [26]. Researchers are attempting to establish mouse-adapted SARS-CoV-2 strains [27, 28] or generate epithelial cytokeratin-18 (K18)-hACE2 transgenic mice to enhance the SARS-CoV-2 infection sensitivity of wild-type mice [29, 30]. However, mouse-adapted SARS-CoV-2 strains are sometimes infective or cause ALI that does not conform to the key characteristics of COVID-19 patients [31, 32], and K18-hACE2 transgenic mice are overreactive in response to SARS-CoV-2 infection due to the enforced exogenous expression of hACE2 [33, 34]. More importantly, live SARS-CoV-2 experiments need to be performed in BSL-3 level laboratories, and such studies have potential biosafety risks.

To confirm the role and illustrate the mechanism of rACE2 in SARS-CoV-2 infection, we chose a mouse model that was coexposed to LPS and the SARS-CoV-2 RBD protein. This mouse model has been applied in studies investigating SARS-CoV [20, 21] and SARS-CoV-2 [35] and can overcome some disadvantages of K18-hACE2 transgenic mice and mouse-adapted SARS-CoV-2 virus discussed above. We found that SARS-CoV-2 RBD protein significantly aggravated LPS-induced ALI as evidenced by a deteriorated pathology, uncontrolled inflammatory response and accumulated oxidative stress. Importantly, rACE2 remarkably ameliorated RBD protein-induced ALI. Mechanistically, the SARS-CoV-2 RBD protein directly binds and downregulates ACE2 and consequently elevates AngI and AngII, further increasing the NOX1/2 and their mediated inflammation and oxidative stress through AT_1_R. rACE2 reversed SARS-CoV-2 RBD protein-aggravated LPS-induced ALI by either directly binding SARS-CoV-2 RBD protein or lowering AngI and/or AngII. Our study is the first to provide evidence suggesting that rACE2 is effective treating SARS-CoV-2 RBD protein-aggravated LPS-induced ALI and presents a molecular explanation that the ACE2-AngII-AT_1_R-NOX1/2 axis is associated with SARS-CoV-2 RBD protein-aggravated LPS-induced ALI.

## Materials and methods

### Cell line and reagents

The HEK293T cell line was purchased from the Cell Bank of the Chinese Academy of Sciences (BS-C162884, Shanghai, China) and was cultured in the DMEM (C11995500BT, Gibco) that contained the 10% fetal bovine serum (FBS, S711-001S, LONSA SCIENCE SRL) and penicillin–streptomycin antibiotics (C0222, Beyotime), followed by incubation within the incubator under 37℃ in the presence of 5% CO_2_. The human IgG1-Fc protein (10702-HNAH), SARS-CoV-2 (2019-nCoV) Spike RBD-Fc recombinant protein (HPLC-verified, 40592-V02H) and the recombinant human ACE2 protein (10108-H08H) were purchased from the Sino Biological. Lipopolysaccharides from Escherichia coli O111:B4 (L2630) and bovine serum albumin (BSA, B2064) were purchased from Sigma-Aldrich.

### Transfection

The expression plasmid for human ACE2 was obtained from Genecopoeia (EX-U1285-M02-B) and the SARS-CoV-2 (2019-nCoV) Spike RBD Gene ORF cDNA clone expression plasmid; N-FLAG tag (Codon Optimized) was purchased from Sino Biological (VG40592-NF). HEK 293 T cells were transfected with vehicle vector, ACE2 plasmid or SARS-CoV-2 spike RBD-FLAG plasmid by using DNA transfection reagent (TF20121201, NEOFECT). The plasmid and DNA transfection reagent were diluted in OPTI-MEM (31985-062, Gibco) followed by incubation with HEK293T cells for 48 h. The transfection efficacy was further determined by qPCR or western blot analysis 48 h after the transfection.

### Co-immunoprecipitation (Co-IP)

Co-immunoprecipitation (Co-IP) was performed to verify the binding of SARS-CoV-2 Spike RBD protein and human ACE2 protein in vitro. In brief, HEK293T cells were co-transfected with ACE2 plasmid and SARS-CoV-2 spike RBD-FLAG plasmid for 48 h. The cells were washed by cold PBS twice and then lysed using RIPA lysis buffer (Abcam; Cambridge, MA, USA) containing PMSF (Sigma-Aldrich) and protease inhibitors (A32953, Thermo Fisher). Anti-FLAG antibody (20543-1-AP, Proteintech) or the negative control IgG antibody (B900620, Proteintech) was diluted to the work concentration at 50 µg/mL with binding/wash buffer (1 × PBS + 0.5% Tween-20, pH 7.4) and then added to Protein A/G Magnetic Beads (HY-K0202, MCE). The beads in the tubes were rotated at room temperature for 30 min and washed for four times. Then, cell lysates were incubated with antibody-conjugated beads at room temperature for 1 h. After that, the beads were washed four times and boiled in 2 × SDS loading buffer for 10 min. The denatured samples were then subjected to western blot analysis to detect the interaction between SARS-CoV-2 spike RBD protein and human ACE2.

### Flow cytometry

Flow cytometry was performed to verify the binding of SARS-CoV-2 Spike protein and human ACE2 protein in vitro. In brief, HEK293T cells were transfected with either empty vector or ACE2 plasmid for 48 h. Then, cells were incubated with SARS-CoV-2 spike RBD-Fc recombinant protein (10 µg) or control-Fc protein (10 µg) in PBS buffer at 37℃ for 2 h, then followed by incubation with anti-Human IgG (Fc specific) antibody (I2136-1ML, Sigma, 1:1000) and Donkey Anti-Goat IgG(H + L), FITC conjugate antibody (1:1000; SA00003-3, Proteintech). Native HEK293T cells incubated with SARS-CoV-2 spike RBD-Fc followed by anti-Fc antibody were shown as controls. After washing cells with PBS, the cells were analyzed by CytoFLEX Flow Cytometry (CytoFLEX S, BECKMAN COULTER). In brief, the live cells were gated according to the values of forward scatter (FSC) and side scatter (SSC). Then, the values of FL1 channel were recorded to determine the intensity of Human IgG (Fc)-FITC. The results were analyzed by FlowJo software.

### Animal handling

**Fig. 1:**
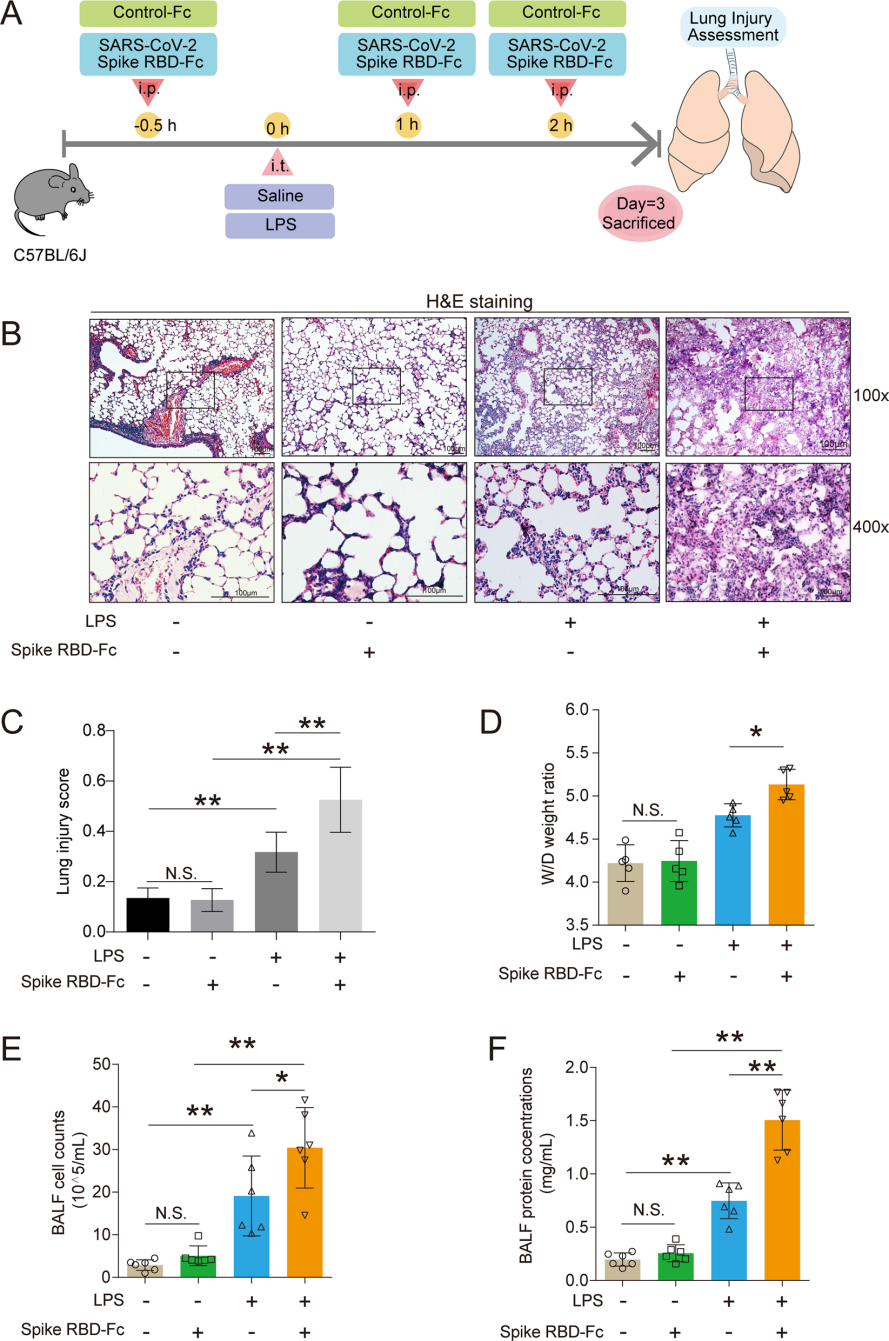
SARS-CoV-2 spike RBD protein enhanced the severity of LPS-induced ALI. **A** The study design for the current study. Male C57BL/6J mice were intratracheally administrated with saline or LPS (0.5 mg/kg). SARS-CoV-2 spike RBD-Fc (5.5 nmol/kg) or equivalent Control-Fc was intraperitoneally injected three times (at 30 min before and at 1 and 2 h after) during LPS treatment. The lung tissues were obtained 3 d after LPS treatment for further assessment of the severity of ALI. **B**. The representative images of H&E staining of the lung tissues. The upper panel, 100×, scale bar = 100 μm; the bottom panel, 400×, scale bar = 100 μm. **C**. The lung injury score was calculated according to the results of H&E staining. (40 fields for each sample were determined.) **D**. The wet-to-dry weight ratio of the lung tissues was determined (*n* = 5 for each group). **E** The cell counts in the BALF were determined (*n* = 6 for each group). **F** The protein concentrations of the BALF were determined by BCA (*n* = 6 for each group). Data are shown as mean ± SD, *P < 0.05, **P < 0.01, and N.S. indicates not significant

All animal experiments and procedures were approved by Animal Laboratory Center of Medical University of Chongqing. All experiments were performed on male C57BL/6 J mice, 8–10 weeks of age and weighted 19–21 g, which were purchased from Byrness Weil biotech Ltd. The procedures for the establishment of this animal model were according to previous studies [20, 21]. To determine the acute lung injury induced by joint exposure to SARS-CoV-2 Spike RBD and LPS, mice were randomly assigned to one of four groups: (1) i.t. Saline + i.p. Control-Fc; (2) i.t. Saline + i.p. SARS-CoV-2 spike RBD-Fc; (3) i.t. LPS + i.t. Control-Fc; and (4) i.t. LPS + i.p. SARS-CoV-2 spike RBD-Fc. In detail, mice were intratracheally administrated with saline or LPS (0.5 mg/kg). SARS-CoV-2 spike RBD-Fc (5.5 nmol/kg) or equivalent Control-Fc was intraperitoneally injected three times (at 30 min before and at 1 and 2 h after) during LPS treatment. The lung tissues were obtained 3 d after LPS treatment for further assessment of the severity of acute lung injury (depicted in Fig. 1A). For the rescue experiments, mice were randomly assigned to one of the two groups: (1) i.t. LPS + i.p. SARS-CoV-2 spike RBD-Fc + i.p. BSA; (2) i.t. LPS + i.p. SARS-CoV-2 spike RBD-Fc + i.p. recombinant human ACE2 protein. In detail, male C57BL/6 J mice were intratracheally administrated with LPS (0.5 mg/kg). SARS-CoV-2 spike RBD-Fc (5.5 nmol/kg) was intraperitoneally injected three times (at 30 min before and at 1 and 2 h after) during LPS treatment. Recombinant human ACE2 protein (1 mg/kg) or equivalent control BSA was intraperitoneally injected 30 min prior to the administration of LPS. The lung tissues were obtained 3 days after LPS treatment for further assessment of the severity of acute lung injury (depicted in Fig. 7A).

The procedures of intratracheal administration were described previously [36]. Briefly, mice were anesthetized using 1% pentobarbital sodium by intraperitoneal injection. After anesthesia, the mice were placed on their back on a 40° slope, and the trachea was intubated using a BD insyte catheter (Franklin Lakes, NJ, USA) with a shortened needle. A total 50 μL normal saline or LPS was instilled followed by 150 μL air using a syringe to ensure the uniformly distribution of LPS in the lung tissues of mice. The animals were held on the head up until regular respiratory rate was observed. Then, the mice were gently moved to the 37℃ heating plate until they recovered from anesthesia.

### Bronchoalveolar lavage fluid (BALF) detection

After indicated treatment, the lung tissues were injected with pre-warmed saline and BALF was carefully collected. The protein concentrations in the supernatant of BALF were determined by bicinchoninic acid method (#5000205, Bio-Rad). Thereafter, the collected BALF was immediately centrifuged for 10 min at 2,000 g at 4℃. The cell pellets from the centrifuged BALF were resuspended in 1 mL saline. The total number of cells in BALF was counted using TC20™ Automated Cell Counter (Bio-Rad).

### Lung edema assessment

The isolated lung tissues were put into the micro-centrifugal tubes, and the wet weight of the lung tissues was measured. Then, the lung tissues were dried in an oven at 60℃ for 48 h, and the dry weights were measured. The wet-to-dry weight ratio of the lung tissues was calculated as the mass of wet lung divided by the mass of dry lung.

### Hematoxylin and eosin (H&E) staining

The pathological changes of lung tissues were subjected to H&E staining according to previous description [37]. Briefly, lung tissues were taken at day 3 after treatment and fixed in 10% formaldehyde. Then, the tissues were sectioned and embedded in paraffin. Hematoxylin–eosin staining of the lung was performed with paraffin slices according to standard protocols. The paraffin sections were deparaffinized in xylene and thereafter dehydrated with ethanol. Then, the slices were incubated with hematoxylin and eosin (D006, Nanjing Jiancheng Bioengineering Institute) at room temperature. The according images were obtained by a microscope (Olympus IX53, Tokyo, Japan).

To calculate the lung injury scores according to the results of H&E staining, 40 random high-power fields (400× total magnification) were independently scored in a blinded fashion for each condition. The selection of random fields typically includes enough random displacements (each at least one high-power field in length) from the current observation position. The features of lung injury, including neutrophils in the alveolar space, neutrophils in the interstitial space, hyaline membranes, proteinaceous debris filling the airspaces and alveolar septal thickening, were determined and weighted according to the relevance ascribed to each feature and then were normalized to the number of fields evaluated. As such, the lung injury score was a continuous value between zero and one (inclusive) [38].

### Immunohistochemical (IHC) analysis

Immunohistochemical assay was conducted according to the protocols described previously [37]. In brief, the lungs of mice were embedded in paraffin and cut into 5 μm sections slides after fixation. Then, section slides were submerged in citric acid buffer and microwaved for 10 min to expose the antigens. 3% hydrogen peroxide was used to quench endogenous peroxidase. 10% goat serum was used to reduce nonspecific binding. Section slides were incubated with antibodies against Fc (Sigma-Aldrich, 12136, 1:100), ACE (Abcam, ab244222, 1:100), ACE2 (Abcam, ab108252, 1:100), CD68 (Proteintech, 28058-1-AP, 1:100) or OGG1 (Proteintech, 15125-1-AP, 1:100), then following incubation with corresponding second antibody. 3,3′-diaminobenzidine (DAB, Solarbio, DA1015) was used to show positive cells. The sections were then observed under a microscope (Olympus, IX53, Tokyo, Japan). The cells with brown color indicated the positive cells for indicated antibody.

### Enzyme-linked immunosorbent assay (ELISA)

The concentrations of IL-6 (EMC004, NeoBioscience), IL-1β (900-M47, NeoBioscience), TNF-α (EM102a, NeoBioscience), MPO (QZ-10367, Jiubang Biology) in the BALF and Angiotensin I (Ang I) (ADI-900-203, Enzo) and Angiotensin II (Ang II) (ADI-900-204, Enzo) in the serum were measured by ELISA. The ELISA was performed in accordance with the manufacturer's instructions. Briefly, the cell-free BALF was obtained and then stored at − 80℃ until the tests were done. The ELISA kits were placed at room temperature for at least 20 min to balance the temperature before use. The specimens or standard substances of different concentrations were added and incubated for 90 min at 37℃. After washing, biotinylated antibody working fluid or diluent was added and incubated for 60 min at 37°C. Repeated washing, the enzyme-conjugated working solution or diluent was incubated at 37℃ for another 20 min out of light. Subsequently, after washing the board, chromogenic substrate was incubated for 15 min at 37℃ in the dark, and the termination solution is then added. The absorbance was measured by a microplate reader (Molecular Devices Corp; Sunnyvale, CA, USA) at wavelength of 450 nm, and the value of indicated factor was calculated.

### Western blot analysis

Western blot analysis was performed according to the previous procedure [39, 40]. Briefly, total protein was extracted from the cell or the lung tissues with RIPA buffer (ab156034, Abcam) containing PMSF (Sigma-Aldrich) and protease inhibitors (A32953, Thermo Fisher). About 30 μg denatured protein sample was subjected to sodium dodecyl sulfate polyacrylamide gel electrophoresis, and then, the protein was transferred to polyvinylidene fluoride membrane (#1620177, Bio-Rad).The membrane was sealed with 5% skim milk (1172GR500, BioFroxx) before incubated with specific primary antibodies at 4℃ overnight, and then, the membrane and horseradish peroxidase-conjugated secondary antibodies (SA00001-1, Proteintech; SA00001-2, Proteintech) were incubated at room temperature for 1 h. The Molecular Imager Gel Doc XR System (Bio-Rad, Hercules, CA, USA) with enhanced chemiluminescence reagents (KGP1128, KeyGEN BioTECH) visualizes the bands, and the Image J software (NIH, Bethesda, MD, USA) was used to analyze bands intensities. The detailed antibody information was given below: ACE, 1:1,000 (ab244222, Abcam); ACE2, 1:800 (ab108252, Abcam); MPO, 1:1000 (22225-1-AP, Proteintech); IκBα, 1:1000 (48145, Cell Signaling Technology); p-IκBα, 1:1000 (28595, Cell Signaling Technology); AKT, 1:1000 (A5031, Bimake); p-AKT, 1:1000 (A5030, Bimake); p-ERK1/2, 1:1,000 (A5036, Bimake); p-p70S6K, 1:1000 (97596, Cell Signaling Technology); HO-1, 1:1000 (27282-1-AP, Proteintech); BIP, 1:1000 (11587-1-AP, Proteintech); p62/SQSTM1, 1:1000 (18420–1-AP, Proteintech); GCLM, 1:1000 (14241-1-AP, Proteintech); TXN, 1:1000 (14999-1-AP, Proteintech); NOX1, 1:1000 (17772-1-AP, Proteintech); NOX2, 1:1000 (A5351,bimake); Vinculin, 1:5000 (66305, Proteintech); β-actin, 1:20,000 (66305, Proteintech); and DYKDDDDK Tag (FLAG) recombinant antibody (Binds To FLAG® Tag Epitope) (1:2000, Proteintech).

### Real-time quantitative PCR (qPCR) analysis

Total RNA was isolated with the qPCR Kits (LS1040, Promega, GoTaq® qPCR and RT-qPCR Systems), following the manufacturer’s instructions. cDNAs were synthesized by using a RT Master Mix for qPCR (HY-K0510, MCE). Real-time PCR was performed with SYBR Green qPCR Master Mix (HY-K0523, MCE). In each reaction, 5 μL of SYBR Green qPCR Master Mix was mixed with 1μL of cDNA sample, 0.2 μL of former primer, 0.2 μL of reverse primer and 3.6 μL of nuclease-free water. RT-qPCR reactions were subjected with CFX Connect real-time system (Bio-Rad, Hercules, CA, USA) using the reaction conditions specified for this assay. The relative gene expression levels were calculated by the 2 (−ΔΔ*Ct*) method and Δnormalized to the expression of GADPH or TATA-binding protein (TBP). Fold change relative to the mean value was determined by 2 (−ΔΔ*Ct*). All the primers were synthesized by Sangon (Shanghai, China), and the sequences are listed in Table 1.

**Table 1:** The sequences of the primers used for qPCR analysis

Gene		Primer sequences
Human ACE2	Forward	5'-GATAGTGGTTGGCATTGTC-3'
Human ACE2	Reverse	5'-CGATGGAGGCATAAGGAT -3'
Human TBP	Forward	5'-ATCAGTGCCGTGGTTCGT-3'
Human TBP	Reverse	5'-TTCGGAGAGTTCTGGGATTG-3'
Mouse p22phox	Forward	5'-CATCAAGCAACCACCTAC -3'
Mouse p22phox	Reverse	5'-CATCTGTCACTGGCATTG -3'
Mouse p47phox	Forward	5'-ACACCTTCATTCGCCATA -3'
Mouse p47phox	Reverse	5'-CTGTAGACCACCTTCTCC -3'
Mouse p67phox	Forward	5'-TTGAAGAAGGGCAGTGAT -3'
Mouse p67phox	Reverse	5'-TTAGGAGGTGGTGGAATATC -3'
Mouse Rac1	Forward	5'-TCATCAGTTACACGACCAA-3'
Mouse Rac1	Reverse	5'-CGCAATCTGTCATAATCTTCT-3'
Mouse Rac2	Forward	5'-ATGTGATGGTGGACAGTAA-3'
Mouse Rac2	Reverse	5'-TAGCGAGAAGCAGATGAG-3'
Mouse NOX1	Forward	5'-GGTGTCTTGCTTGATAATCTT-3'
Mouse NOX1	Reverse	5'-CTTGGTCGTTCTATGTTGTT-3'
Mouse NOX2	Forward	5'-CAAGAACATTACCTTCCACAT-3'
Mouse NOX2	Reverse	5'-TGATATAGCCAGTCCAACC-3'
Mouse NOX3	Forward	5'-GTGTGGTATAAGTGTTGTGAA-3'
Mouse NOX3	Reverse	5'-TAAATGAGCCTTCCCTTGT-3'
Mouse NOX4	Forward	5'-TCAGTGAACTACAGTGAAGAT-3'
Mouse NOX4	Reverse	5'-GGAATGGTTGTGAAGAGAAG-3'
Mouse NOX5	Forward	5'-TACTACCTGGACATCACTAAC-3'
Mouse NOX5	Reverse	5'-CTTGGAGAATGGCTAGGAT-3'
Mouse Duox1	Forward	5'-TTCCTCAACCGCTACATT-3'
Mouse Duox1	Reverse	5'-GTGGACTGATGGAGAACA-3'
Mouse Duox2	Forward	5'-GAAGACTGCTCAAGATGTG-3'
Mouse Duox2	Reverse	5'-TCAATCACTATCCTGTCCTC-3'
Mouse TBP	Forward	5'-ATCAGTGCCGTGGTTCGT-3'
Mouse TBP	Reverse	5'-TTCGGAGAGTTCTGGGATTG-3'
Mouse GAPDH	Forward	5'-AACCTGCCAAGTATGATGA-3'
Mouse GAPDH	Reverse	5'-GGAGTTGCTGTTGAAGTC-3'

### Statistical analysis

The data are shown as the mean ± standard deviation (S.D.). Comparisons between two groups were made by unpaired Student's *t* test or Mann–Whitney U test. One-way analysis of variance (ANOVA) followed by least significant difference (LSD) *t* test or Kruskal–Wallis test was used to compare the significant differences among groups. All statistical tests were conducted using Prism 9.0 software (GraphPad Software, San Diego, CA, USA). *P < 0.05 and **P < 0.01 were considered as significant, and N.S. indicated not significant.

## Results

### SARS-CoV-2 spike RBD protein enhanced the severity of LPS-induced ALI

**Fig. 2:**
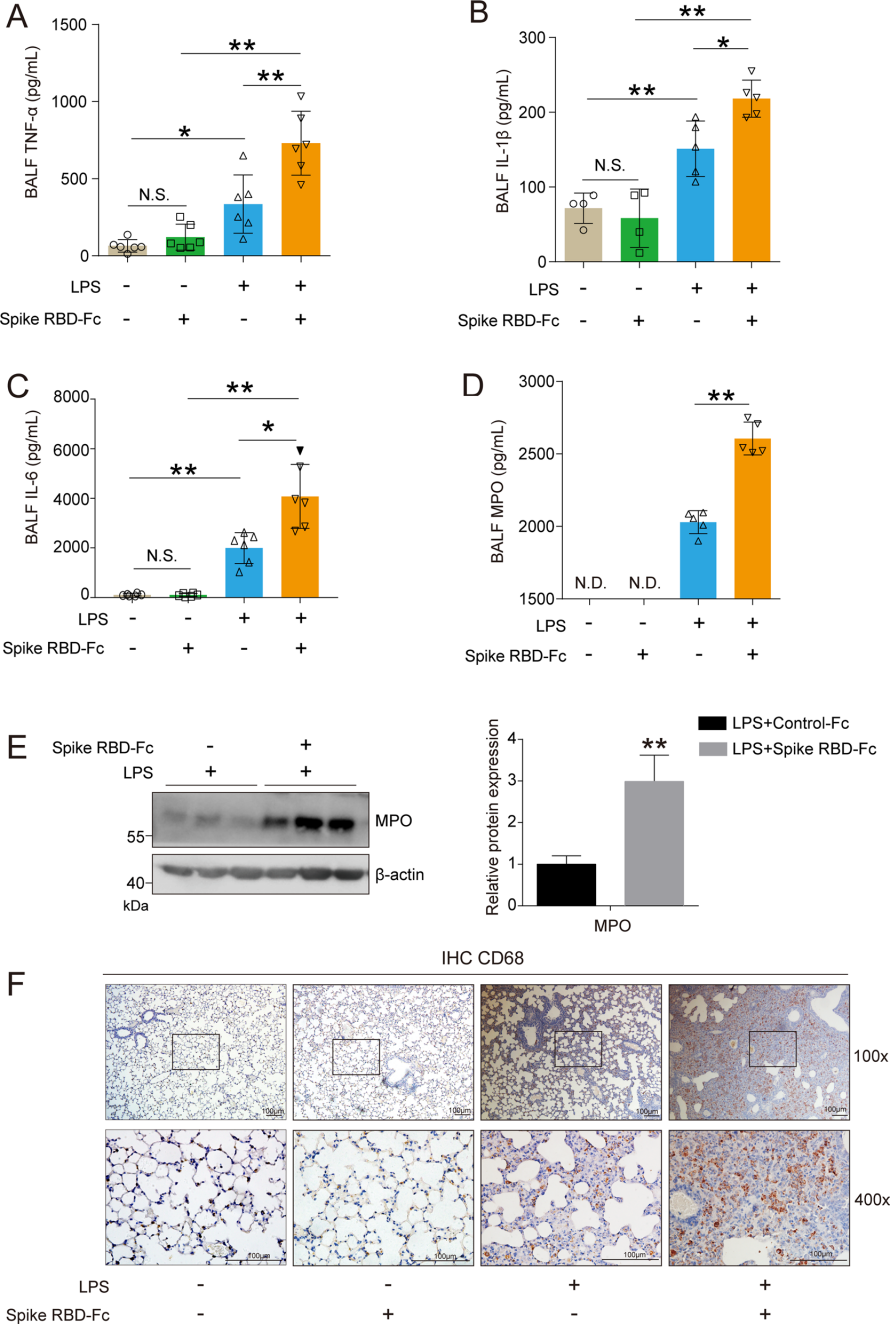
SARS-CoV-2 spike RBD protein enhanced the severity of LPS-induced inflammation in the lung tissues. The proinflammatory cytokines (**A**). TNF-α, **B** IL-1β and (**C**) IL-6 in the BALF were determined by ELISA (*n* = 4–6 for each group). **D** The concentrations of MPO in the BALF were determined by ELISA (*n* = 5 for each group). N.D. indicates not detectable. **E** The protein expression levels of MPO were determined by western blot analysis. **F** The macrophage infiltration in the lung tissues was determined by IHC, and the representative images were shown. CD68 was used as the maker for the staining of macrophage. The upper panel, 100×, scale bar = 100 μm; the bottom panel, 400×, scale bar = 100 μm. Data are shown as mean ± S.D., *P < 0.05, **P < 0.01, and N.S. indicates not significant

To assess whether SARS-CoV-2 spike RBD protein was able to aggravate LPS-induced lung injury, male SPF C57BL/6 J mice were randomly assigned to the following groups: (1) i.t. Saline + i.p. Control-Fc; (2) i.t. Saline + i.p. SARS-CoV-2 spike RBD-Fc; (3) i.t. LPS + i.t. Control-Fc; and (4) i.t. LPS + i.p. SARS-CoV-2 spike RBD-Fc. The detailed information of the animal treatments is described in the Materials and Methods section. The lung tissues of the mice from indicated groups were obtained and used to detect the severity of acute lung injury 3 d after instillation (Fig. 1A). The H&E staining results showed that either SARS-CoV-2 spike RBD-Fc protein or Control-Fc protein induced unapparent histopathological changes in the lung tissues. As expected, LPS caused moderate lung injury, including infiltration of inflammatory cells and thickened alveolar walls. Strikingly, SARS-CoV-2 spike RBD protein significantly aggravated the LPS-induced lung pathological deterioration compared with Control-Fc protein. Disruption of the alveolar structure was prevalent in the mouse lung tissues coexposed to SARS-CoV-2 spike RBD protein and LPS (Fig. 1B). Correspondingly, the SARS-CoV-2 spike RBD protein profoundly increased the lung injury score (Fig. 1C). Noncardiogenic pulmonary edema is a hallmark of ALI/ARDS [41]. The lung tissue weight–dry weight ratios are good indicators for the assessment of lung edema. Our results clearly indicated that the SARS-CoV-2 spike RBD protein markedly increased LPS-induced lung edema (Fig. 1D). Bronchoalveolar lavage fluid (BALF) is commonly used to determine local tissue damage and inflammation in lung tissues. Our results indicated that the LPS treatment induced an elevation in the cell counts and protein concentrations in the BALF, while SARS-CoV-2 spike RBD protein further led to elevations of cell counts and protein concentrations in the BALF (Fig. 1E and F). Uncontrolled inflammatory responses or inflammatory cytokine storms are crucial events during ALI/ARDS [42]. We found that SARS-CoV-2 spike RBD protein enhanced the LPS-induced elevation in proinflammatory cytokines in the BALF, including TNF-α (Fig. 2A), IL-1β (Fig. 2B) and IL-6 (Fig. 2C). As expected, MPO (a marker of neutrophil activation) was further elevated in the BALF (Fig. 2D) and lung tissues (Fig. 2E). Moreover, the infiltration of CD68-positive macrophages was more prevalent in lung tissues of the mice treated with SARS-CoV-2 spike RBD protein and LPS (Fig. 2F). Collectively, although the instillation of SARS-CoV-2 spike RBD protein alone is unable to induce a significant inflammatory response and ALI, SARS-CoV-2 spike RBD protein significantly enhances LPS-induced ALI.

### SARS-CoV-2 spike RBD protein directly bind ACE2

**Fig. 3:**
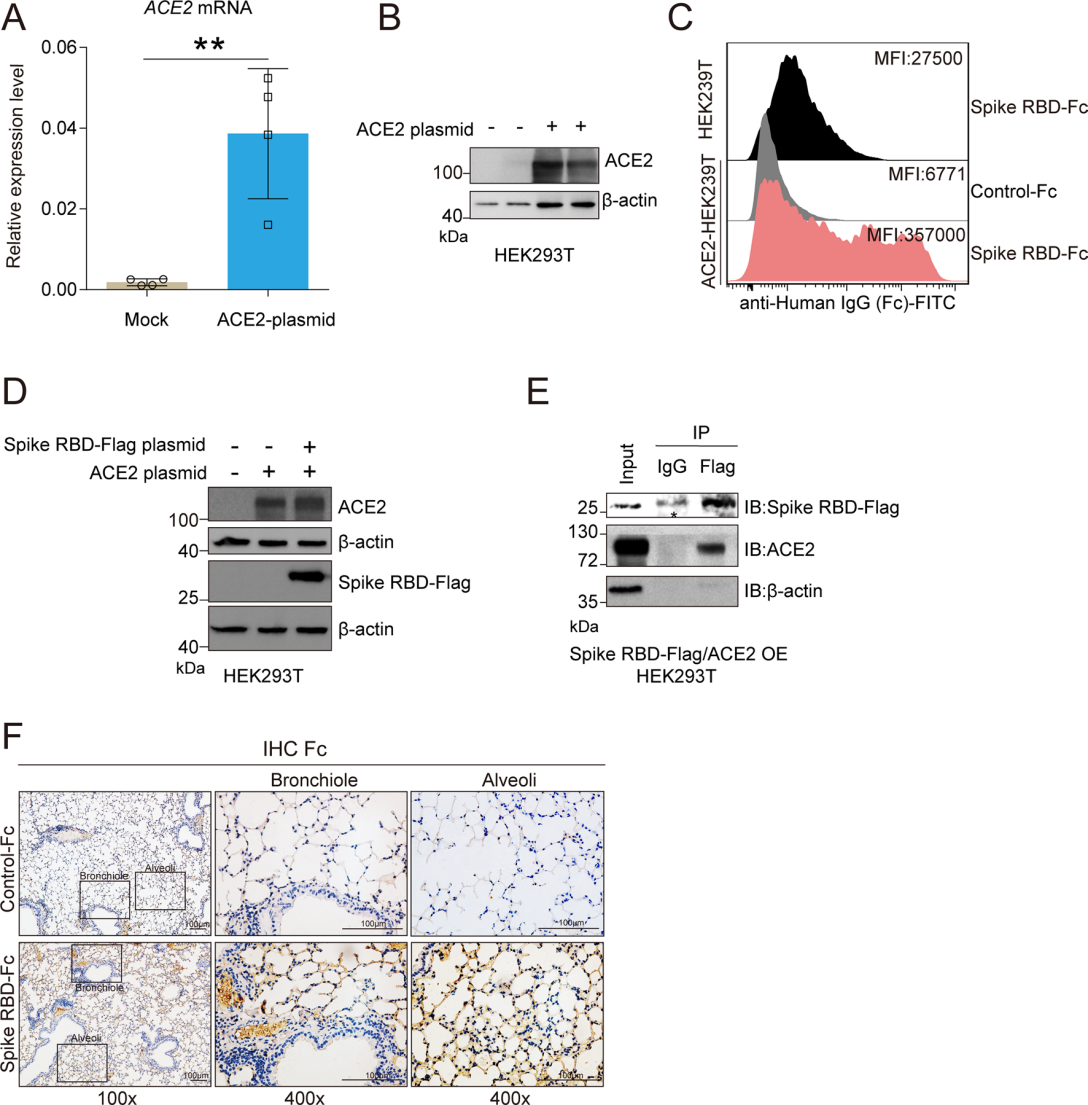
SARS-CoV-2 spike RBD protein binds ACE2. ACE2 plasmid or empty vector was transiently transfected into HEK293T cells for 48 h. **A** The mRNA expression levels of ACE2 were determined by qPCR analysis (*n* = 4 for each group). **B** The protein expression levels of ACE2 were determined by western blot analysis, and the representative images were shown. **C** HEK293T cells transfected with ACE2 were incubated with Control-Fc protein or SARS-CoV2 Spike RBD-Fc protein, and the binding was detected by FACS using anti-Fc antibody (indicated by gray or pink shadow). Native HEK293T cells incubated with Spike-Fc followed by anti-Fc antibody were shown as controls (indicated by black shadow). The median fluorescence intensity (MFI) for each group was shown. **D** HEK239T cells were transfected with ACE2 plasmid, SARS-CoV-2 spike RBD-Flag plasmid or both of them for 48 h. The protein expression levels of ACE2 and Flag were determined by western blot analysis, and the representative images were shown. **E** HEK239T cells were co-transfected with ACE2 plasmid and SARS-CoV-2 spike RBD-Flag plasmid for 48 h. Co-immunoprecipitation analysis using anti-Flag antibody was performed to demonstrate the binding of ACE2 and SARS-CoV-2 spike RBD-Flag, and the representative images were shown. * indicates the light chain of anti-Flag antibody. **F** The expression level of Fc in the lung tissues of mice followed by injection of Control-Fc or SARS-CoV-2 spike RBD-Fc was determined by IHC, and the representative images were shown. Data are shown as mean ± S.D., **P < 0.01

SARS-CoV spike protein can bind and downregulate ACE2, which is recognized as a key event involved in SARS-CoV-induced lung injury [20, 21, 43]. Here, we confirmed that SARS-CoV-2 spike protein binds ACE2 directly in vitro. The endogenous expression of ACE2 protein in HEK293T cells is almost undetectable. We used an ACE2 plasmid to enforce the overexpression of ACE2 protein in HEK293T cells, and the qPCR and western blot analyses results indicated that the ACE2 plasmid transfection led to a remarkable elevation in ACE2 in comparison with the vector plasmid transfection at the transcriptional (Fig. 3A) and translational levels (Fig. 3B), respectively. The FACS results indicated that SARS-CoV-2 spike RBD-Fc protein, but not Control-Fc protein, could effectively bind ACE2 in HEK293T cells (Fig. 3C). We further constructed a co-transfection HEK293T cell model with a SARS-CoV-2 spike RBD-Flag plasmid and ACE2 plasmid. The western blot analysis results suggested that the co-transfection strategy was successful in increasing the expression of both SARS-CoV-2 spike RBD-Flag and ACE2 in HEK293T cells (Fig. 3D). The Co-IP analysis results indicated that ACE2 directly binds SARS-CoV-2 spike RBD-Flag (Fig. 3E). In addition, we found that the instillation of SARS-CoV-2 spike RBD protein-Fc, but not Control-Fc protein, could prominently deposit in the bronchioles and alveoli in mice (Fig. 3F), which was likely the basis of the direct interaction between SARS-CoV-2 spike RBD protein and endogenous ACE2 in vivo.

### SARS-CoV-2 spike RBD protein decreased ACE2 and increased the AngII levels

**Fig. 4:**
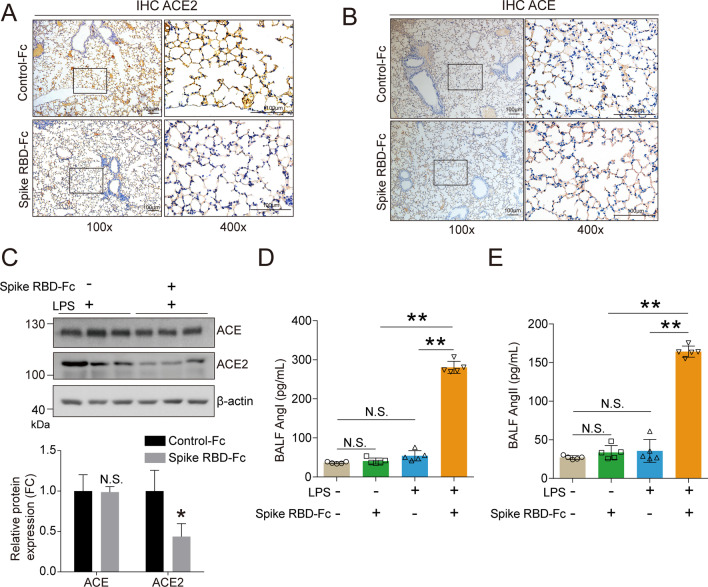
SARS-CoV-2 spike RBD protein led to dysfunction of the RAS system in mice. The expression level of (**A**). ACE2 and (**B**). ACE in the lung tissues of mice followed by injection of Control-Fc or SARS-CoV-2 spike RBD was determined by IHC, and the representative images were shown. The left panel, 100×, scale bar = 100 μm; the right panel, 400×, scale bar = 100 μm. Mice were treated with saline or LPS combined by Control-Fc or SARS-CoV-2 spike RBD-Fc for 3 days. (**C**). The protein expression levels of ACE and ACE2 in the lung tissues were determined by western blot analysis, and the representative results were shown. (**D**). Angiotensin I (Ang I) and (**E**). Angiotensin II (Ang II) concentrations in the BALF were determined by ELISA analysis (*n* = 5–6 per group). Data are shown as mean ± S.D., *P < 0.05, **P < 0.01, and N.S. indicates not significant

We further investigated the biological effects induced by the instillation of SARS-CoV-2 spike RBD protein. The IHC analysis results suggested that SARS-CoV-2 spike RBD-Fc protein led to a decrease in ACE2 in the lung tissues (Fig. 4A), but had a negligible influence on the ACE (Fig. 4B). The western blot analysis results confirmed that SARS-CoV-2 spike RBD-Fc protein, but not Control-Fc protein, caused a decrease in ACE2 and had minimal influence on ACE in the lung tissues of mice treated with LPS (Fig. 4C). Interestingly, we found that although Control-Fc protein, SARS-CoV-2 spike RBD-Fc protein and LPS did not induce a significant change in AngI or AngII, co-exposure to SARS-CoV-2 spike RBD-Fc protein and LPS caused a remarkable increase in AngI and AngII in the BALF by an ELISA analysis (Fig. 4D, E).

### SARS-CoV-2 spike RBD protein preferentially activated AngII-AT_1_R-NF-κB-NOX1/2 signaling

**Fig. 5:**
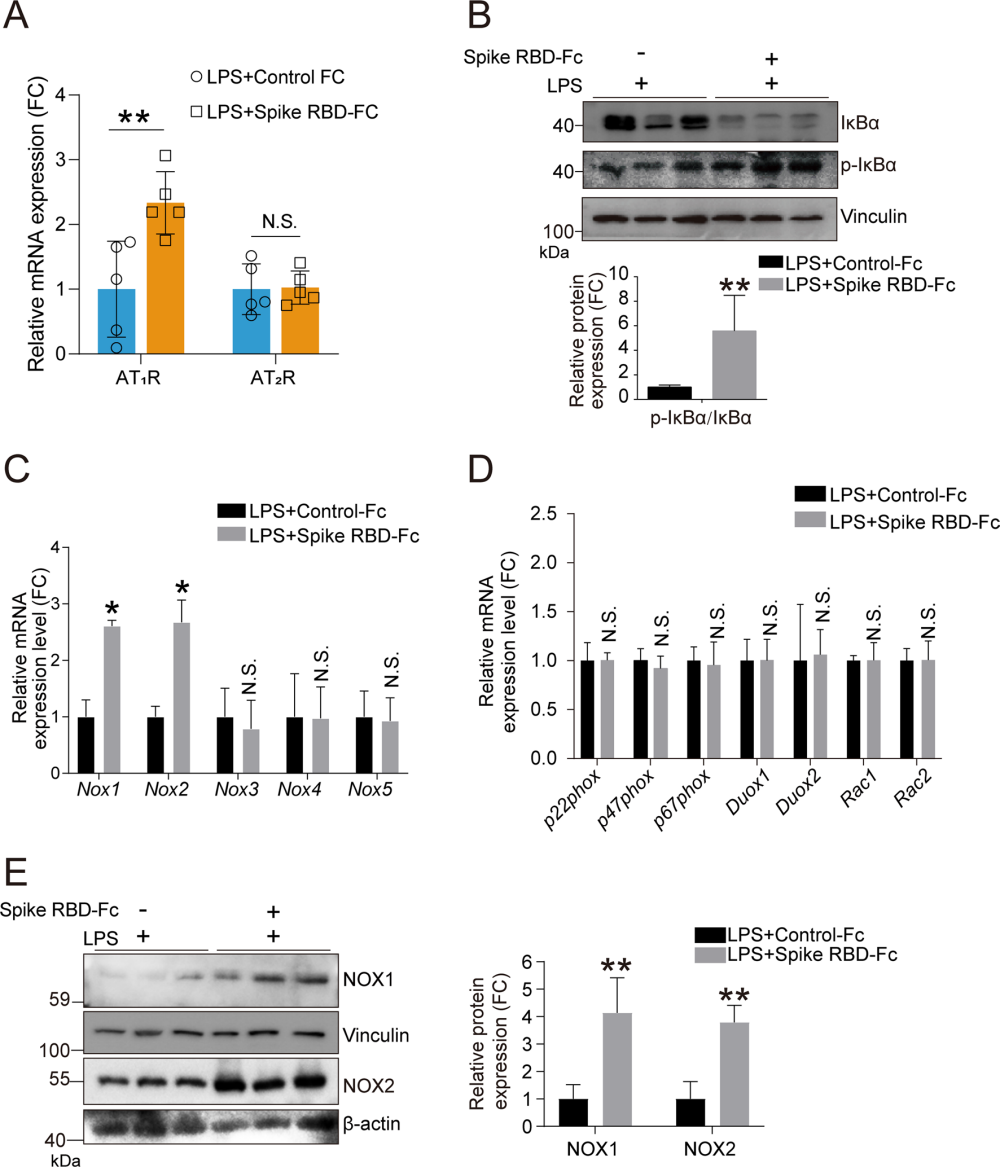
SARS-CoV-2 spike RBD protein activated the NOX1 and NOX2. Mice were exposed to LPS with or without SARS-CoV-2 spike RBD-Fc protein for 3 days. **A** The mRNA expression levels of AT_1_R and AT_2_R in the lung tissues of mice with indicated treatment were determined by qPCR analysis (*n* = 5 per group). **B** The protein expression levels of IκB and p-IκB in the lung tissues were determined by western blot analysis, and the representative results were shown. **C, D** The mRNA expression levels of indicated gene in the lung tissues were determined by qPCR analysis (*n* = 5 per group). **E** The protein expression levels of NOX1 and NOX2 in the lung tissues were determined by western blot analysis, and the representative results were shown. Data are shown as mean ± S.D., *P < 0.05, **P < 0.01, and N.S. indicates not significant

Subsequently, we determined the downstream effects of elevated AngII. The qPCR analysis results indicated increased mRNA expression of AT_1_R but not AT_2_R, suggesting that elevated AngII likely activated AT_1_R in the lung tissues of mice treated with SARS-CoV-2 spike RBD-Fc protein and LPS (Fig. 5A). LPS is a classical activator of NF-κB signaling by binding TLR4 [44]. The western blot analysis showed that the SARS-CoV-2 spike RBD protein enhanced the increase in the ratio of p-IκBα/total IκBα (Fig. 5B), suggesting further activation of NF-κB signaling. Persistent elevation of AngII promotes the pathogenesis of several diseases via oxidative stress and the induction of inflammatory cytokines [45, 46]; particularly SARS-CoV-2 enhanced LPS-induced ALI. NOX/DUOX NADPH oxidases are widely expressed and serve as key producers of ROS in mammalian cells with intricate regulation [47, 48]. Thus, we determined the mRNA expression levels of the NADPH oxidases family. Intriguingly, we found that only the NOX1 and NOX2 mRNA expression levels were significantly elevated by SARS-CoV-2 spike RBD-Fc protein compared with Control-Fc protein when coexposed to LPS (Fig. 5C, D). Correspondingly, the protein expression levels of NOX1 and NOX2 were profoundly elevated (Fig. 5E). Altogether, the above results indicate that SARS-CoV-2 spike RBD protein might preferentially activate AngII-AT_1_R-NF-κB-NOX1/2 signaling.

### SARS-CoV-2 spike RBD protein led to the aggravation of oxidative stress and inflammation

**Fig. 6:**
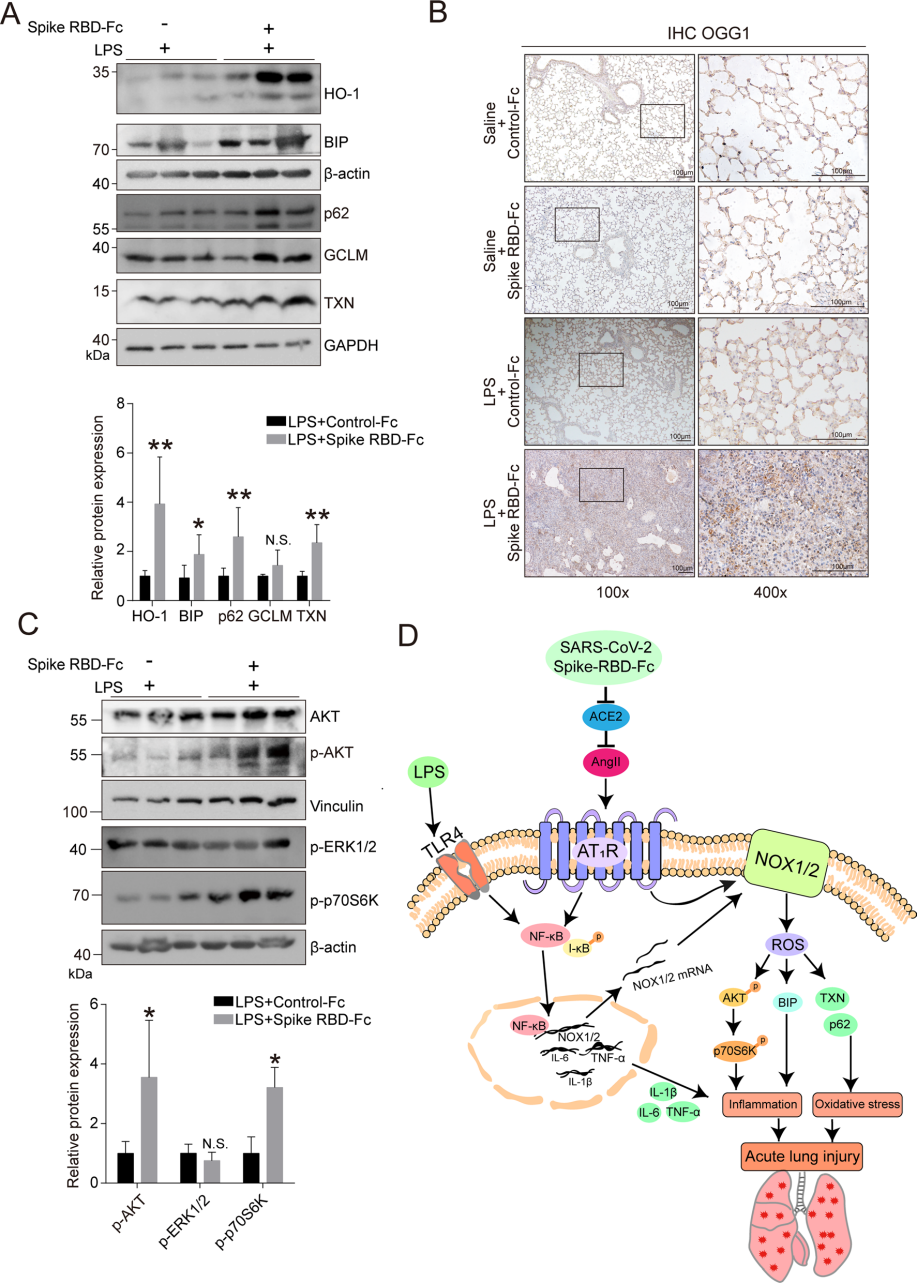
SARS-CoV-2 spike RBD protein enhanced oxidative stress in LPS-induced ALI. Mice were exposed to LPS with or without SARS-CoV-2 spike RBD-Fc protein for 3 days. **A** The protein expression levels of HO-1, BIP, p62, GCLM and TXN in the lung tissues were determined by western blot analysis, and the representative results were shown. **B** The OGG1 expression level in the lung tissues was determined by IHC, and the representative images were shown. OGG1 was used as the maker for the staining of oxidative stress. The left panel, 100×, scale bar = 100 μm; the right panel, 400×, scale bar = 100 μm. **C** The protein expression levels of AKT, p-AKT, p-ERK1/2 and p-p70S6K in the lung tissues were determined by western blot analysis, and the representative results were shown. **D** Schematic diagram of SARS-CoV-2 spike RBD protein enhanced severity of LPS-induced acute lung injury. Data are shown as mean ± S.D., *P < 0.05, **P < 0.01, and N.S. indicates not significant

NOX1/2 can control oxidative stress [48]. As anticipated, several antioxidant factors, such as HO-1, BIP, p62 and TXN, were significantly elevated upon SARS-CoV-2 spike RBD protein treatment compared with Control-Fc protein treatment when coexposed to LPS (Fig. 6A). 8-oxoguanine DNA glycosylase (OGG1) is the rate-limiting enzyme and is responsible for the proper removal of 8-hydroxydeoxyguanosine (8-OHdG) from DNA in response to oxidative stress; therefore, OGG1 can be considered as a marker of oxidative stress [49]. The IHC results clearly indicated that OGG1 was significantly elevated in the lung tissues of mice treated with SARS-CoV-2 spike RBD protein and LPS (Fig. 6B). These data suggest that SARS-CoV-2 spike RBD protein led to the aggravation of oxidative stress induced by LPS. NOX1/2-generated ROS can further induce/aggravate inflammation through several cellular signaling transduction pathways, such as AKT or ERK/p38 MAPK signaling [48, 50, 51]. Intriguingly, SARS-CoV-2 spike RBD protein was able to activate AKT signaling as evidenced by an increase in p-AKT and its downstream factor p-p70S6K. In contrast, no apparent change was observed in p-ERK1/2. These data suggest that SARS-CoV-2 spike RBD protein induced NOX1/2-mediated AKT signaling pathway activation (Fig. 6C).

Based on the above observations, we outline a possible mechanism by which SARS-CoV-2 spike RBD protein aggravated LPS-induced ALI as follows: SARS-CoV-2 spike RBD protein directly binds and decreases ACE2, consequently leading to an increase in Ang II. AT_1_R serves as a major receptor for elevated Ang II; on the one hand, AT_1_R facilitates the activation of the NF-κB pathway alone with the LPS treatment; on the other hand, AT_1_R enhances the activation of NOX1/2, subsequently aggravating the inflammatory response via the AKT pathway. Both oxidative stress and inflammatory response are contributed to SARS-CoV-2 RBD protein-induced ALI (Fig. 6D).

### Recombinant ACE2 protein ameliorated SARS-CoV-2 spike RBD protein-aggravated LPS-induced ALI

**Fig. 7:**
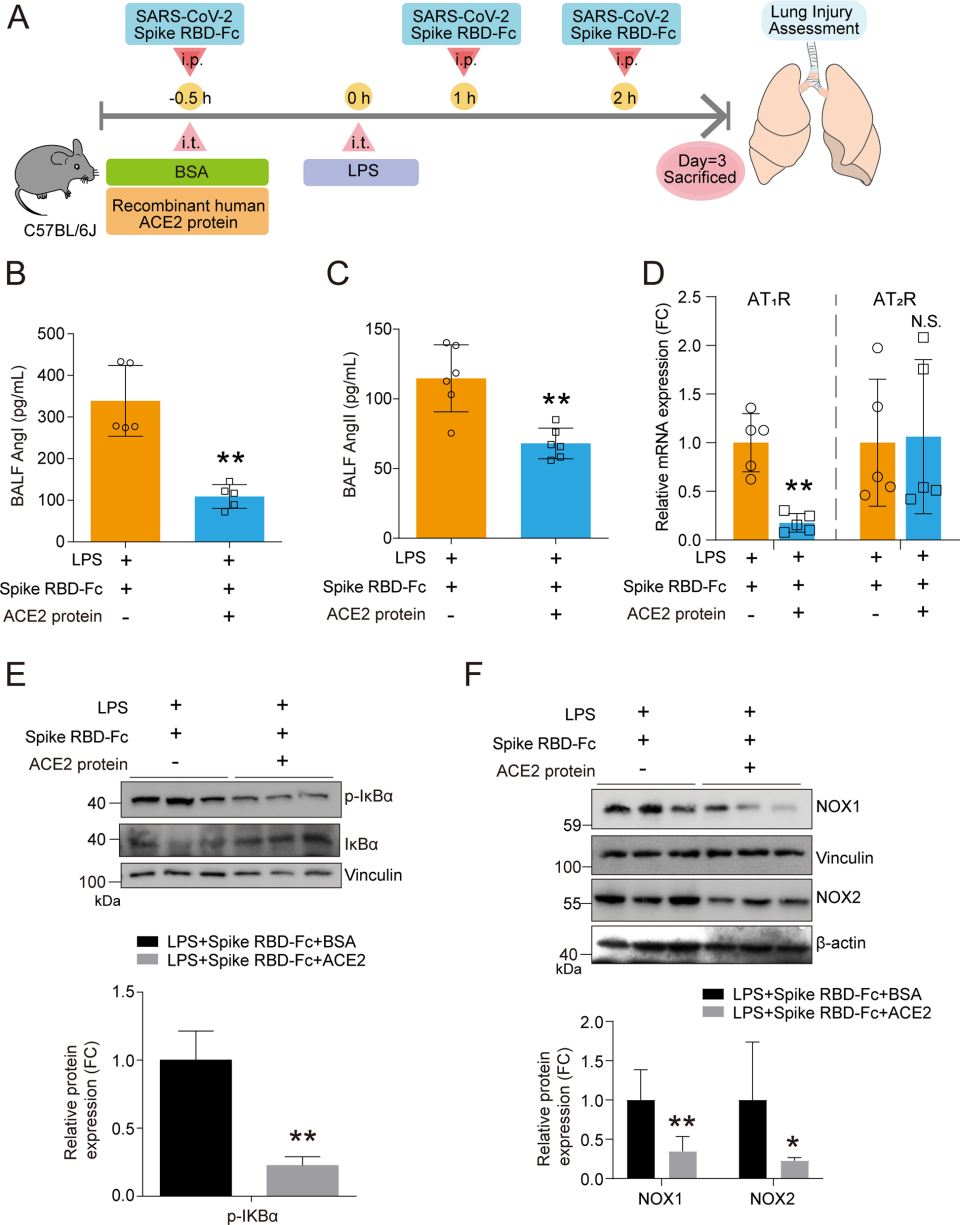
Recombinant ACE2 protein reshaped the profile of the RAS system in ALI induced by coexposed to SARS-CoV-2 spike RBD and LPS. **A** The study design for the ACE2 rescue study. Male C57BL/6 J mice were intratracheally administrated with LPS (0.5 mg/kg). SARS-CoV-2 spike RBD-Fc (5.5 nmol/kg) was intraperitoneally injected three times (at 30 min before and at 1 and 2 h after) during LPS treatment. Recombinant human ACE2 protein (1 mg/kg) or equivalent control BSA was intraperitoneally injected 30 min prior to the administration of LPS. The lung tissues were obtained 3 days after LPS treatment for further assessment of the severity of acute lung injury. **B** Ang I and **C** Ang II concentrations in the BALF were determined by ELISA analysis (*n* = 5–6 per group). **D** The mRNA expression levels of AT1R and AT2R in the lung tissues of mice with indicated treatment were determined by qPCR analysis (*n* = 5 per group). **E–F** The protein expression levels of IκB, p-IκB NOX1 and NOX2 in the lung tissues were determined by western blot analysis, and the representative results were shown. Data are shown as mean ± S.D., **P < 0.01, and N.S. indicates not significant

A key objective of the current study was to assess whether recombinant ACE2 protein (rACE2) could protect against ALI induced by SARS-CoV-2 spike RBD protein. Either rACE2 or BSA was injected intraperitoneally, and the degree of ALI was assessed 3 days after the treatment with SARS-CoV-2 spike RBD protein (Fig. 7A). As anticipated, rACE2 dramatically lowered the elevation in Ang I (Fig. 7B) and Ang II (Fig. 7C) in the BALF and decreased the expression level of AT_1_R, but not AT_2_R, in the lung tissues (Fig. 7D). Consequently, the western blot analysis showed that rACE2 decreased the expression levels of p-IκB/IκB (Fig. 7E) and NOX1 and NOX2 (Fig. 7F).

**Fig. 8:**
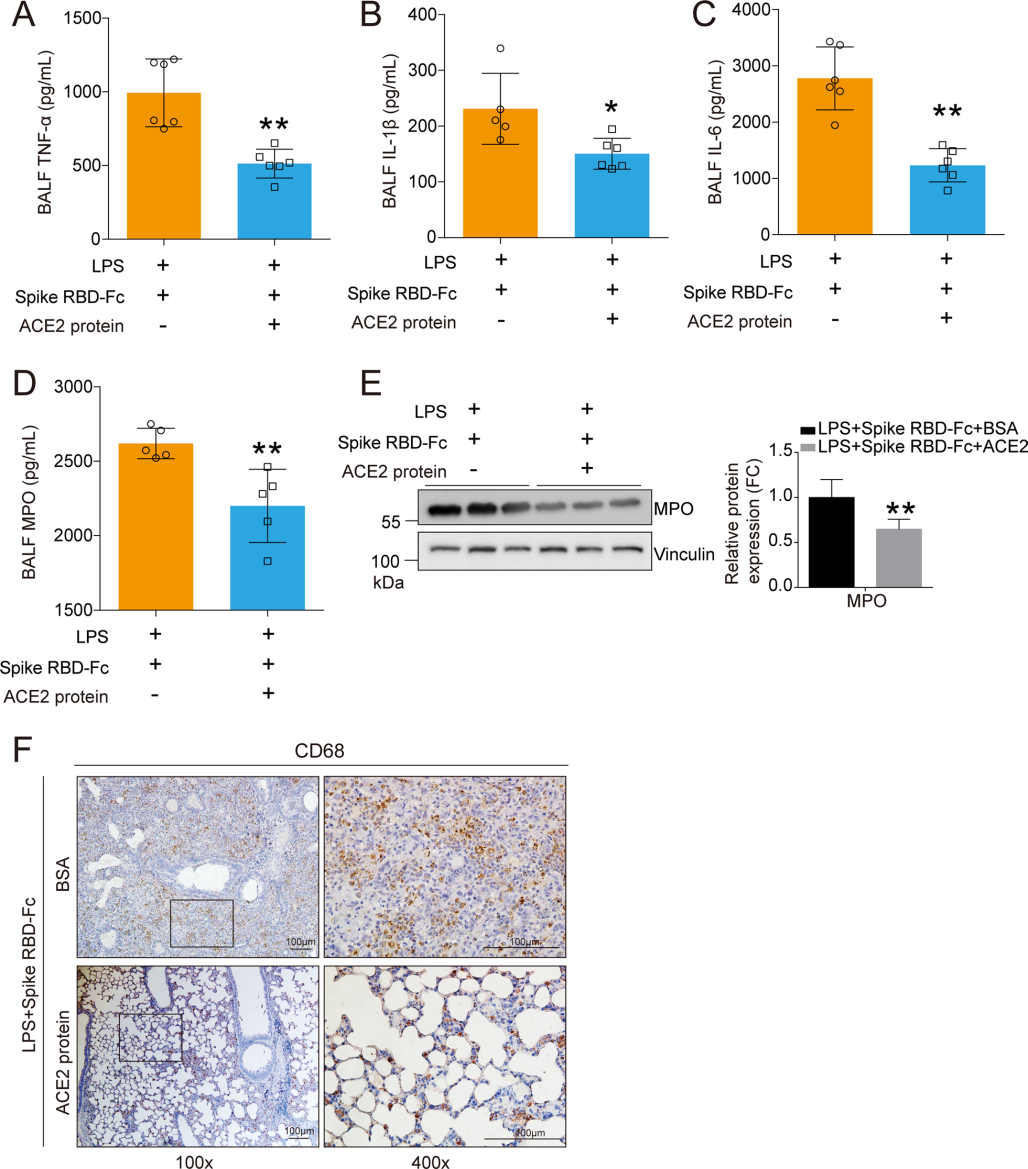
Recombinant ACE2 protein decreased the inflammation induced by coexposed to SARS-CoV-2 spike RBD and LPS. The proinflammatory cytokines **A** TNF-α, **B** IL-1β and **C** IL-6 in the BALF were determined by ELISA (*n* = 5–6 for each group). **D** The concentrations of MPO in the BALF were determined by ELISA (*n* = 5 for each group). N.D. indicates not detectable. **E** The protein expression levels of MPO were determined by western blot analysis, and the representative results were shown. **F** The macrophage infiltration in the lung tissues was determined by IHC, and the representative images were shown. CD68 was used as the maker for the staining of macrophage. The left panel, 100×, scale bar = 100 μm; the right panel, 400×, scale bar = 100 μm. Data are shown as mean ± S.D., *P < 0.05 and **P < 0.01

**Fig. 9:**
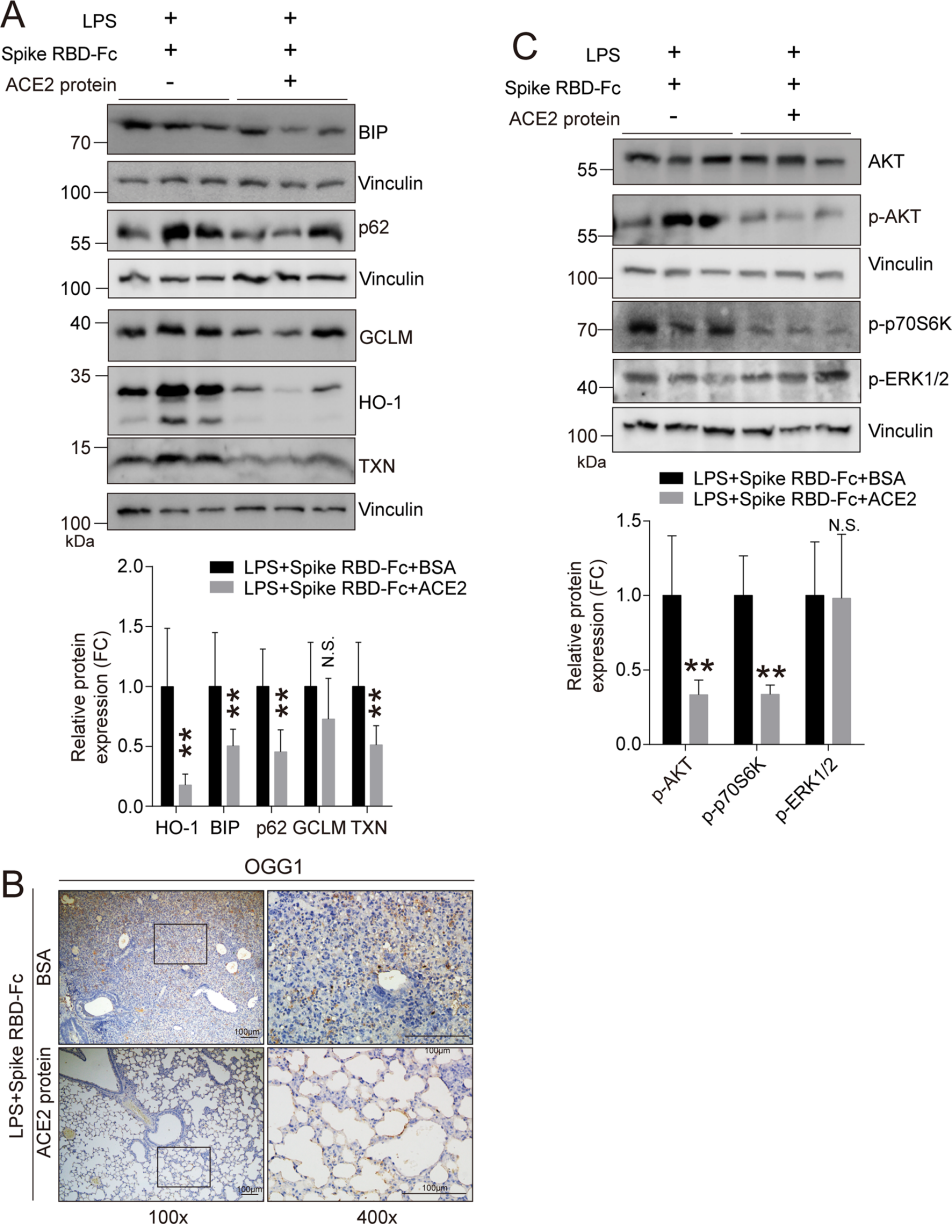
Recombinant ACE2 protein reduced oxidative stress induced by coexposed to SARS-CoV-2 spike RBD and LPS. **A** The protein expression levels of HO-1, BIP, p62, GCLM and TXN in the lung tissues were determined by western blot analysis, and the representative results were shown. **B** The OGG1 expression level in the lung tissues was determined by IHC, and the representative images were shown. OGG1 was used as the maker for the staining of oxidative stress. The left panel, 100 × , scale bar = 100 μm; the right panel, 400×, scale bar = 100 μm. **C** The protein expression levels of AKT, p-AKT, p-ERK1/2 and p-p70S6K in the lung tissues were determined by western blot analysis, and the representative results were shown. Data are shown as mean ± S.D., **P < 0.01, and N.S. indicates not significant

We further determined whether the inflammatory responses were restrained by rACE2. All data from the BALF and the lung tissues supported our hypothesis that rACE2 could control the inflammatory responses, including the decreased release of the proinflammatory factors TNF-α (Fig. 8A), IL-1β (Fig. 8B) and IL-6 (Fig. 8C) and MPO activity (Fig. 8D) in the BALF and the reduced expression of the MPO protein (Fig. 8E) and CD68-positive macrophages in the lung tissues (Fig. 8F). Antioxidative stress factors including HO-1, BIP, p62, GCLM and TXN (Fig. 9A) and oxidative stress marker OGG1 (Fig. 9B) were decreased in the lung tissues of mice treated with rACE2, indicating that oxidative stress was sharply suppressed by rACE2. Additionally, the AKT pathway, the downstream signaling pathway triggered by SARS-CoV-2 spike RBD protein, was significantly decreased by rACE2. However, the ERK pathway was not obviously influenced (Fig. 9C).

**Fig. 10:**
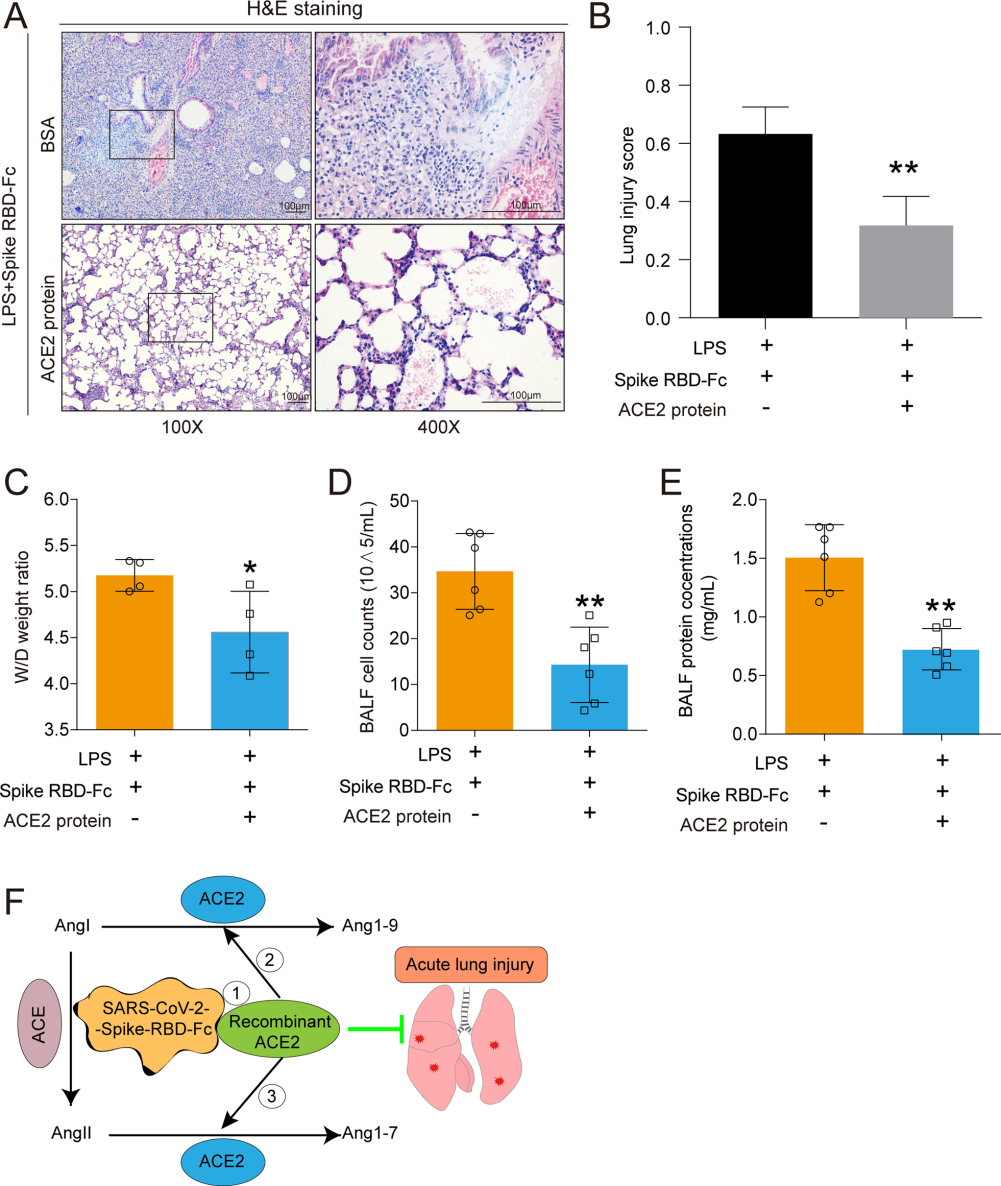
Recombinant ACE2 protein ameliorated ALI induced by coexposed to SARS-CoV-2 spike RBD and LPS. **A** The representative images of H&E staining of the lung tissues. The left panel, 100 × , scale bar = 100 μm; the right panel, 400×, scale bar = 100 μm. **B** The lung injury score was calculated according to the results of H&E staining. (40 fields for each sample were determined.) **C** The wet-to-dry weight ratio of the lung tissues was determined (*n* = 4 for each group). **D** The cell counts in the BALF were determined (*n* = 6 for each group). **E** The protein concentrations of the BALF were determined by BCA (n = 6 for each group). **F** Schematic diagram of the mechanism of the rescue effects of rACE2. Data are shown as mean ± S.D., *P < 0.05 and **P < 0.01

Importantly, the histopathological results showed that rACE2 sufficiently mitigated the alveolar damage induced by SARS-CoV-2 spike RBD protein compared with BSA (Fig. 10A) and decreased the lung injury scores according to the H&E staining results (Fig. 10B). Consistently, rACE2 profoundly decreased the lung edema (Fig. 10C), total cell counts in the BALF (Fig. 10D) and protein exudation in the BALF (Fig. 10E).

Collectively, we propose the following potential roles of rACE2 in the treatment of SARS-CoV-2-induced ALI: 1) rACE2 directly binds SARS-CoV-2 spike RBD protein, thereby reducing the binding of SARS-CoV-2 spike RBD protein at endogenous ACE2; 2) rACE2 cleaves AngI to generate Ang1-9, thereby suppressing the conversion of AngI to AngII; and 3) rACE2 cleaves AngII to generate Ang1-7, thereby directly decreasing the levels of AngII (Fig. 10F).

## Discussion

A large observational study suggested that ACE-inhibitor (ACEI)/Angiotensin II receptor blocker (ARB) therapy at least did not increase susceptibility to COVID-19 or worsen the outcome of COVID-19 patients [52]. Another observational study reported that the inpatient use of ACEI/ARB was linked to a lower risk of all-cause mortality, suggesting that ACEI/ARB therapy has the potential to improve the outcome of COVID-19 patients [53]. Most strikingly, in a case report, human recombinant soluble ACE2 protein was administered to a 45-year-old woman with severe COVID-19 for 7 days, and reduced AngII concentrations and cytokines in the plasma were observed. On day 57, the patient was discharged from the hospital, supporting the potential application of rACE2 as clinical intervention for SARS-CoV-2 infection [24]. However, the conclusions of retrospective cohort studies concerning the effects of ARB/ACEI drugs for the treatment of SARS-CoV-2 infection are controversial, because some studies found no association of mortality or severity in COVID-19 patients taking ACEI/ARB [54]. To completely address the above concerns, clinical trials have been initiated to demonstrate the causal link between the ACE2-AngII axis and SARS-CoV-2 infection-induced ALI/ARDS. A randomized controlled trial (RCT) phase 2 study investigating rACE2 therapy in 181 participants is currently ongoing (ClinicalTrials.gov, Identifier: NCT04335136). Moreover, ongoing RCTs evaluating ACEI/ARB (NCT04312009/NCT04311177/NCT04329195) are expected to shed more light on the current disputation.

Nevertheless, targeting the AngII-ACE2 axis to develop effective therapy is currently a hot topic for researchers [55]. Corresponding strategies targeting AngII and ACE2 have been developed. RAS imbalance contributed to a swine model as evidenced by the fact that infusing AngII or blocking ACE2 induced increased pulmonary damage, while the infusion of an ARB and low-molecular-weight heparin ameliorated pulmonary damage, which might reflect the features of the pathogenesis of COVID-19 [56]. Linsky et al. described a de novo design strategy that allowed them to engineer decoy proteins, namely CTC-445, that bind the spike protein of SARS-CoV-2 by replicating the hACE2 interface [57]. The SARS-CoV-2 load was sharply reduced by rACE2 up to 5000-fold in cell experiments and engineered organoids, directly demonstrating that SARS-CoV-2 can be effectively neutralized by ACE2 [58]. Monteil et al. demonstrated that rACE2 enhanced the effect of remdesivir on Vero E6 and kidney organoids and proposed combinatorial regimens for the treatment of COVID-19 [59]. Engineered ACE2 receptor traps were developed to neutralize SARS-CoV-2 in cells [60]. More recently, soluble ACE2 was shown to have excellent imbibition effects on all current variants of concern, including the Alpha, Beta, Gamma and Delta, and several variants of interest in Vero E6 cells and human lung epithelial cells [61]. In addition, the same research team reported that the intranasal administration of aerosolized soluble recombinant human ACE2 profoundly repressed weight loss and promoted animal survival in a BALB/c mouse model infected with a mouse-adapted SARS-CoV-2 strain [62].

Dysfunction of the RAS system, particularly uncontrolled elevation of AngII and decreased ACE2 are linked to ALI induced by SARS-CoV virus infection [20, 21, 43], high pathological avian influenza virus infection (H5N1/H7N9) [22, 23] and, recently, SARS-CoV-2 virus infection [24, 58]. Our study substantially supplements the current knowledge regarding the link between AngII-ACE2 axis and the pathogenesis of severe pulmonary deterioration in SARS-CoV-2-induced pneumonia. The increases in AngI and AngII in the BALFs and the enhanced lung injury induced by the combination of SARS-CoV-2 spike RBD protein and LPS in the current study represent an alternative model to avoid unnecessary exposure to a live SARS-CoV-2 and share some features of live SARS-CoV-2. Here, we demonstrated that SARS-CoV-2 spike RBD protein binds ACE2 in vitro, is directly deposited in the alveolus of mice and aggravates LPS-induced ALI. Importantly, we further indicated that rACE2 played an excellent protective role in coping with SARS-CoV-2 spike RBD protein-induced ALI, including markedly suppressing the inflammatory response in lung tissues and BALFs and mitigating histopathological deterioration. While we are preparing this manuscript, another research group reported that soluble ACE2 protein inhalation protected mice from a mouse-adapted SARS-CoV-2 infection-induced pulmonary immunopathology [62]. The above research and our study are mutually confirmatory, suggesting that rACE2 is effective as a treatment for SARS-CoV-2-induced ALI in vivo by either intraperitoneal injection or intranasal inhalation.

Notably, a multinational, double-blind RCT (ATHOS-3) involving 163 patients with vasodilatory shock demonstrated that AngII possesses vasopressor effects [63]. AngII directly facilitates apoptosis and the skeletal reconstruction of pulmonary microvascular endothelial cells, subsequently accelerating the disruption of the pulmonary microvascular endothelial barrier and leading to more pulmonary exudate and edema [64], which likely share the characteristics of the pathological manifestations of COVID-19 patients and, therefore, are assumed to be linked to the pathogenesis of COVID-19. Mechanistically, AngII functions as a harmful factor in lung homeostasis by binding and activating AT_1_R, further leading to an excessive inflammatory response and eventually lung injury [65, 66]. Our study supports the above notion and proves that SARS-CoV-2 spike RBD protein-induced elevation of AngII mediated lung injury through AT_1_R but not AT_2_R.

We preliminarily revealed the reciprocal activation of oxidative stress and the inflammatory response and pointed out that NOX1 and/or NOX2 might be critical regulators involved in SARS-CoV-2 spike RBD protein-induced ALI. On the one hand, AngII stimulated AT_1_R and then promoted LPS-triggered NF-κB activation, followed by the vast transcriptional activation of proinflammatory cytokines, including TNF-α, IL-6 and Il-1β (Fig. 2A–C), which might originate from infiltrating macrophages. Our IHC results support this notion (Fig. 2F). Notably, NF-κB signal transduction could induce the transcriptional activation of NOX1 and NOX2 mRNA (Fig. 5C), which contributed to the elevations of NOX1 and NOX2 proteins in lung tissues (Fig. 5E); on the other hand, AngII-AT_1_R signal transduction could contribute to oxidative stress through NOX1 and NOX2, which is likely specifically caused by SARS-CoV-2 spike RBD protein according to our qPCR results (Fig. 5C). It has been reported that AngII induces the accumulation of ROS (superoxide and hydrogen peroxide) produced by activated NADPH oxidase, which are causally linked to various pathophysiological processes [66, 67]. Indeed, we observed a marked elevation in oxidative stress-associated factors BIP, HO-1, TXN and p62 (Fig. 6A) along with OGG1 elevation (Fig. 6B). These results indicate a burst of ROS in SARS-CoV-2 spike RBD protein-induced ALI.

The dysregulation of NOX signaling is likely associated with the main COVID-19 comorbidities, including hypertension, cardiovascular diseases, obesity and diabetes [68]. NOXs are crucial regulators responsible for the production of ROS in the vascular system. It is believed that NOXs-driven intravascular ROS is important for vascular dysfunction in response to hypertension. Because the loss of endothelial function is a common feature in COVID-19 patients with coexisting cardiovascular diseases, it is assumed that NOXs play a crucial role in the pathogenesis of complications of SARS-CoV-2 infection [69]. The relationship between NOXs activation and the severity of COVID-19 has been recently reported [70]. The value of soluble Nox2-derived peptide (sNox2-dp), a serum marker of NOX2 activation, was significantly elevated in COVID-19 patients (*n* = 182) compared to that in the controls, and the value of sNox2-dp was positively correlated with the severity of COVID-19 [70]. Our study is the first to provide the evidence in an animal model showing that NOX1 and/or NOX2 activation in lung tissue might be associated with SARS-CoV-2 spike RBD protein-induced ALI. The value of targeting NOX1/2 in the treatment for SARS-CoV-2 infection-induced ALI/ARDS should be determined in the future.

AngII-AT_1_R-NOX1/2 activation causes the ROS generation, further influencing various downstream signaling targets of Ang II, at least including MAP kinases and AKT kinases [71]. Our results support that SARS-CoV-2 spike RBD protein-induced ROS generation preferentially activated AKT kinases, but not ERK1/2 (Fig. 6C), shielding the means by targeting AKT to cope with SARS-CoV-2-induced ALI. A plausible explanation is that, after the inhibition of AKT, the number of CD4^+^/FoxP3^+^/CD103^+^/CTLA4^+^ effector regulatory T cells (Tregs) in the ARDS lung increases to control the inflammatory response and facilitate the recovery of damaged lung tissues [72, 73]. Therefore, the use of a combination of AKT inhibitors, such as triciribine [74] and MK2206 [75], and antiviral drugs to combat COVID-19 should be considered. However, further experiments are needed to prove this hypothesis.

However, notably, the current study has several limitations as follows: (1) The animal model is limited with respect to human pathophysiology; therefore, the results presented here are only suggestive; (2) LPS significantly differs from human acute lung injury and, even in rodents, does not necessarily replicate sepsis- and/or virus-pneumonia-induced ALI; therefore, ongoing studies should consider confirming the conclusion in the current study using a live virus or a sepsis-induced ALI mouse model; and (3) it could be valuable to determine whether the inhibition of AT_1_R or NOX1/2 contributes to the repression of SARS-CoV-2-induced ALI in the future.

Taken together, we demonstrate that SARS-CoV-2 spike RBD protein could aggravate LPS-induced ALI, which can be remarkably ameliorated by rACE2 as rACE2 directly binds SARS-CoV-2 spike RBD protein and, thus, prevents the virus from binding the original cellular ACE2 receptor; moreover, rACE2 cleaves AngI and/or AngII independent of its binding SARS-CoV-2 spike RBD protein. We highlight the importance of the Ang II/AT_1_R/NOX1/2 axis-mediated ROS burst in the uncontrolled inflammatory response in SARS-CoV-2 spike RBD protein-induced ALI and suggest that rACE2 can be applied to the clinical treatment of COVID-19 alone or combined with other antiviral agents.

## Data Availability

Data and materials may be made available upon written request to the corresponding author.

## Abbreviations

8-OHdG: 8-Hydroxydeoxyguanosine
ALI: Acute lung injury
ARDS: Acute respiratory distress syndrome
ACE: Angiotensin-converting enzyme
ACE2: Angiotensin-converting enzyme 2
ARB: Angiotensin receptor blockers
AngI: Angiotensin I
AngII: Angiotensin II
Ang1-7: Angiotensin 1–7
Ang1-9: Angiotensin 1–9
AT_1_R: Angiotensin II receptor type 1
AT_2_R: Angiotensin II receptor type 2
BSA: Bovine serum albumin
BALF: Bronchoalveolar lavage fluid
COVID-19: Coronavirus disease 2019
Co-IP: Co-immunoprecipitation
cDNAs: Complementary DNAs
CTLA4: Cytotoxic T-lymphocyte-associated protein 4
DMEM: Dulbecco’s Modified Eagle Medium
DAB: 3:3′-Diaminobenzidine
ERK1/2: Extracellular signal-regulated kinase
DUOX: Dual oxidase
ELISA: Enzyme-linked immunosorbent assay
FBS: Fetal bovine serum
FITC: Fluorescein isothiocyanate
FACS: Fluorescence-activated cell sorting
FoxP3: Forkhead box P3
GCLM: Glutamate–cysteine ligase modifier subunit
GADPH: Glyceraldehyde-3-phosphate dehydrogenase
hACE2: The human version of ACE2
HEK293T: Human embryonic kidney cells 293
H&E: Hematoxylin and eosin
HO-1: Heme Oxygenase 1
IgG: Immunoglobulin G
IHC: Immunohistochemistry
IL-6: Interleukin-6
IL-1β: Interleukin-1β
IκBα: Inhibitory protein κB
LPS: Lipopolysaccharide;
MPO: Myeloperoxidase
MAPK: Mitogen-activated protein kinase
NOX1: NADPH oxidase 1
NOX2: NADPH oxidase 2
NADPH: Nicotinamide adenine dinucleotide phosphate
NF-κB: Nuclear factor kappa-light-chain-enhancer of activated B cells
OGG1: 8-Oxoguanine DNA glycosylase
PMSF: Phenylmethanesulfonylfluoride
PBS: Phosphate-buffered saline
p-p70S6K: Phospho-p70 ribosomal S6 kinase
p62/SQSTM1: Protein p62/Sequestosome 1
PCR: Polymerase chain reaction
RT-qPCR: Real-time quantitative PCR
ROS: Reactive oxygen species
RIPA: Radio-immunoprecipitation assay
RAS: Renin–angiotensin system
RBD: Receptor binding domains
RCT: Randomized controlled trial
rACE2: Recombinant ACE2 protein
SARS-CoV: Severe acute respiratory syndrome coronavirus
SARS-CoV-2: Severe acute respiratory syndrome coronavirus 2
SDS: Sodium dodecyl sulfate
SPF: Specific-pathogen-free
sNox2-dp: Soluble Nox2-derived peptide
TMPRSS2: Transmembrane protease serine 2
TNF-α: Tumor necrosis factor-α
TXN: Thioredoxin
TBP: TATA-binding protein
TLR4: Toll-like receptor 4

## References

[1] Badawi S, Ali BR (2021). ACE2 nascence, trafficking, and SARS-CoV-2 pathogenesis: the saga continues. Hum Genomics.

[2] Cheng H, Wang Y, Wang GQ (2020). Organ-protective effect of angiotensin-converting enzyme 2 and its effect on the prognosis of COVID-19. J Med Virol.

[3] Gheblawi M, Wang K, Viveiros A (2020). Angiotensin-converting enzyme 2: SARS-CoV-2 receptor and regulator of the renin-angiotensin system: celebrating the 20th anniversary of the discovery of ACE2. Circ Res.

[4] Wang Q, Zhang Y, Wu L (2020). Structural and functional basis of SARS-CoV-2 entry by using human ACE2. Cell.

[5] Reinke LM, Spiegel M, Plegge T (2017). Different residues in the SARS-CoV spike protein determine cleavage and activation by the host cell protease TMPRSS2. PLoS ONE.

[6] Dong M, Zhang J, Ma X (2020). ACE2, TMPRSS2 distribution and extrapulmonary organ injury in patients with COVID-19. Biomed Pharmacother.

[7] Xiong J, Xiang Y, Huang Z (2021). Structure-based virtual screening and identification of potential inhibitors of SARS-CoV-2 S-RBD and ACE2 interaction. Front Chem.

[8] El-Arif G, Farhat A, Khazaal S et al. The Renin-Angiotensin system: a key role in SARS-CoV-2-induced COVID-19. Molecules 2021; 26. 10.3390/molecules26226945 PMC8622307 34834033

[9] D'Amico S, Tempora P, Lucarini V et al. ERAP1 and ERAP2 enzymes: a protective shield for RAS against COVID-19? Int J Mol Sci 2021; 22. 10.3390/ijms22041705 PMC7914632 33567739

[10] Perini MV, Dmello RS, Nero TL (2020). Evaluating the benefits of renin-angiotensin system inhibitors as cancer treatments. Pharmacol Ther.

[11] Renziehausen A, Wang H, Rao B (2019). The renin angiotensin system (RAS) mediates bifunctional growth regulation in melanoma and is a novel target for therapeutic intervention. Oncogene.

[12] Flores-Munoz M, Smith NJ, Haggerty C (2011). Angiotensin1-9 antagonises pro-hypertrophic signalling in cardiomyocytes via the angiotensin type 2 receptor. J Physiol.

[13] Wang K, Liu X, Xiao H (2017). The correlation between inflammatory injury induced by LPS and RAS in EpH4-Ev cells. Int Immunopharmacol.

[14] Dang Z, Su S, Jin G (2020). Tsantan Sumtang attenuated chronic hypoxia-induced right ventricular structure remodeling and fibrosis by equilibrating local ACE-AngII-AT1R/ACE2-Ang1-7-Mas axis in rat. J Ethnopharmacol.

[15] Seltzer S (2020). Linking ACE2 and angiotensin II to pulmonary immunovascular dysregulation in SARS-CoV-2 infection. Int J Infect Dis.

[16] Liu Y, Yang Y, Zhang C (2020). Clinical and biochemical indexes from 2019-nCoV infected patients linked to viral loads and lung injury. Sci China Life Sci.

[17] Wu Z, Hu R, Zhang C (2020). Elevation of plasma angiotensin II level is a potential pathogenesis for the critically ill COVID-19 patients. Crit Care.

[18] Osman IO, Melenotte C, Brouqui P (2021). Expression of ACE2, soluble ACE2, angiotensin I, angiotensin II and angiotensin-(1–7) is modulated in COVID-19 patients. Front Immunol.

[19] van Lier D, Kox M, Santos K et al. Increased blood angiotensin converting enzyme 2 activity in critically ill COVID-19 patients. ERJ Open Res 2021; 7. 10.1183/23120541.00848-2020 PMC7848790 33738305

[20] Imai Y, Kuba K, Rao S (2005). Angiotensin-converting enzyme 2 protects from severe acute lung failure. Nature.

[21] Kuba K, Imai Y, Rao S (2005). A crucial role of angiotensin converting enzyme 2 (ACE2) in SARS coronavirus-induced lung injury. Nat Med.

[22] Zou Z, Yan Y, Shu Y (2014). Angiotensin-converting enzyme 2 protects from lethal avian influenza A H5N1 infections. Nat Commun.

[23] Huang F, Guo J, Zou Z (2014). Angiotensin II plasma levels are linked to disease severity and predict fatal outcomes in H7N9-infected patients. Nat Commun.

[24] Zoufaly A, Poglitsch M, Aberle JH (2020). Human recombinant soluble ACE2 in severe COVID-19. Lancet Respir Med.

[25] Abd El-Aziz TM, Al-Sabi A, Stockand JD (2020). Human recombinant soluble ACE2 (hrsACE2) shows promise for treating severe COVID-19. Signal Transduct Target Ther.

[26] Damas J, Hughes GM, Keough KC (2020). Broad host range of SARS-CoV-2 predicted by comparative and structural analysis of ACE2 in vertebrates. Proc Natl Acad Sci USA.

[27] Muruato A, Vu MN, Johnson BA (2021). Mouse-adapted SARS-CoV-2 protects animals from lethal SARS-CoV challenge. PLoS Biol.

[28] Dinnon KH, Leist SR, Schafer A (2020). A mouse-adapted model of SARS-CoV-2 to test COVID-19 countermeasures. Nature.

[29] Yinda CK, Port JR, Bushmaker T (2021). K18-hACE2 mice develop respiratory disease resembling severe COVID-19. PLoS Pathog.

[30] Dong W, Mead H, Tian L et al. The K18-hACE2 transgenic mouse model recapitulates non-severe and severe COVID-19 in response to infectious dose of SARS-CoV-2 virus. J Virol 2021:JVI0096421. 10.1128/JVI.00964-21 PMC8754221 34668775

[31] Chu H, Chan JF (2021). A lethal mouse model using a mouse-adapted SARS-CoV-2 strain with enhanced binding to mouse ACE2 as an important platform for COVID-19 research. EBioMedicine.

[32] Leist SR, Dinnon KH, Schafer A (2020). A mouse-adapted sars-cov-2 induces acute lung injury and mortality in standard laboratory mice. Cell.

[33] Zhang L, Chen S, Zhang W (2021). An update on animal models for severe acute respiratory syndrome coronavirus 2 infection and countermeasure development. Front Microbiol.

[34] Kumari P, Rothan HA, Natekar JP et al. Neuroinvasion and encephalitis following intranasal inoculation of SARS-CoV-2 in K18-hACE2 mice. Viruses 2021; 13. 10.3390/v13010132 PMC7832889 33477869

[35] Liu T, Wu J, Han C (2021). RS-5645 attenuates inflammatory cytokine storm induced by SARS-CoV-2 spike protein and LPS by modulating pulmonary microbiota. Int J Biol Sci.

[36] Jiang X, Tang Q, Zhang J (2018). Autophagy-dependent release of zinc ions is critical for acute lung injury triggered by zinc oxide nanoparticles. Nanotoxicology.

[37] Qin X, Tang Q, Jiang X (2020). Zinc oxide nanoparticles induce ferroptotic neuronal cell death in vitro and in vivo. Int J Nanomedicine.

[38] Matute-Bello G, Downey G, Moore BB (2011). An official American Thoracic Society workshop report: features and measurements of experimental acute lung injury in animals. Am J Respir Cell Mol Biol.

[39] Qin X, Zhang J, Wang B et al. Ferritinophagy is involved in the zinc oxide nanoparticles-induced ferroptosis of vascular endothelial cells. Autophagy 2021:1–20. 10.1080/15548627.2021.1911016 PMC8726675 33843441

[40] Zhang J, Qin X, Wang B (2017). Zinc oxide nanoparticles harness autophagy to induce cell death in lung epithelial cells. Cell Death Dis.

[41] Thompson BT, Chambers RC, Liu KD (2017). Acute respiratory distress syndrome. N Engl J Med.

[42] Villar J, Ferrando C, Martinez D (2020). Dexamethasone treatment for the acute respiratory distress syndrome: a multicentre, randomised controlled trial. Lancet Respir Med.

[43] Kuba K, Imai Y, Rao S (2006). Lessons from SARS: control of acute lung failure by the SARS receptor ACE2. J Mol Med (Berl).

[44] Imai Y, Kuba K, Neely GG (2008). Identification of oxidative stress and Toll-like receptor 4 signaling as a key pathway of acute lung injury. Cell.

[45] Nickenig G, Harrison DG (2002). The AT(1)-type angiotensin receptor in oxidative stress and atherogenesis: part I: oxidative stress and atherogenesis. Circulation.

[46] Forrester SJ, Booz GW, Sigmund CD (2018). Angiotensin II signal transduction: an update on mechanisms of physiology and pathophysiology. Physiol Rev.

[47] Panday A, Sahoo MK, Osorio D (2015). NADPH oxidases: an overview from structure to innate immunity-associated pathologies. Cell Mol Immunol.

[48] Aviello G, Knaus UG (2018). NADPH oxidases and ROS signaling in the gastrointestinal tract. Mucosal Immunol.

[49] Simone S, Gorin Y, Velagapudi C (2008). Mechanism of oxidative DNA damage in diabetes: tuberin inactivation and downregulation of DNA repair enzyme 8-oxo-7,8-dihydro-2'-deoxyguanosine-DNA glycosylase. Diabetes.

[50] Koundouros N, Poulogiannis G (2018). Phosphoinositide 3-kinase/Akt signaling and redox metabolism in cancer. Front Oncol.

[51] Nakanishi A, Wada Y, Kitagishi Y (2014). Link between PI3K/AKT/PTEN pathway and NOX proteinin diseases. Aging Dis.

[52] Fosbol EL, Butt JH, Ostergaard L (2020). Association of angiotensin-converting enzyme inhibitor or angiotensin receptor blocker use with COVID-19 diagnosis and mortality. JAMA.

[53] Zhang P, Zhu L, Cai J (2020). Association of inpatient use of angiotensin-converting enzyme inhibitors and angiotensin II receptor blockers with mortality among patients with hypertension hospitalized with COVID-19. Circ Res.

[54] Singh R, Rathore SS, Khan H (2021). Mortality and severity in COVID-19 patients on ACEIs and ARBs-a systematic review, meta-analysis, and meta-regression analysis. Front Med (Lausanne).

[55] Vaduganathan M, Vardeny O, Michel T (2020). Renin-angiotensin-aldosterone system inhibitors in patients with Covid-19. N Engl J Med.

[56] Rysz S, Al-Saadi J, Sjostrom A (2021). COVID-19 pathophysiology may be driven by an imbalance in the renin-angiotensin-aldosterone system. Nat Commun.

[57] Linsky TW, Vergara R, Codina N (2020). De novo design of potent and resilient hACE2 decoys to neutralize SARS-CoV-2. Science.

[58] Monteil V, Kwon H, Prado P (2020). Inhibition of SARS-CoV-2 infections in engineered human tissues using clinical-grade soluble human ACE2. Cell.

[59] Monteil V, Dyczynski M, Lauschke VM (2021). Human soluble ACE2 improves the effect of remdesivir in SARS-CoV-2 infection. EMBO Mol Med.

[60] Glasgow A, Glasgow J, Limonta D (2020). Engineered ACE2 receptor traps potently neutralize SARS-CoV-2. Proc Natl Acad Sci USA.

[61] Wirnsberger G, Monteil V, Eaton B et al. Clinical grade ACE2 as a universal agent to block SARS-CoV-2 variants. bioRxiv 2021. 10.15252/emmm.202115230 PMC9350269 35781796

[62] Shoemaker RH, Panettieri RA, Jr., Libutti SK et al. Development of a novel, pan-variant aerosol intervention for COVID-19. bioRxiv 2021.

[63] Khanna A, English SW, Wang XS (2017). Angiotensin II for the treatment of vasodilatory shock. N Engl J Med.

[64] Busse LW, Chow JH, McCurdy MT (2020). COVID-19 and the RAAS-a potential role for angiotensin II?. Crit Care.

[65] Meng Y, Yu CH, Li W (2014). Angiotensin-converting enzyme 2/angiotensin-(1–7)/Mas axis protects against lung fibrosis by inhibiting the MAPK/NF-kappaB pathway. Am J Respir Cell Mol Biol.

[66] Hanna IR, Taniyama Y, Szocs K (2002). NAD(P)H oxidase-derived reactive oxygen species as mediators of angiotensin II signaling. Antioxid Redox Signal.

[67] Griendling KK, Minieri CA, Ollerenshaw JD (1994). Angiotensin II stimulates NADH and NADPH oxidase activity in cultured vascular smooth muscle cells. Circ Res.

[68] Guan WJ, Ni ZY, Hu Y (2020). Clinical characteristics of coronavirus disease 2019 in China. N Engl J Med.

[69] Meza CA, La Favor JD, Kim DH et al. Endothelial dysfunction: is there a hyperglycemia-induced imbalance of NOX and NOS? Int J Mol Sci 2019; 20. 10.3390/ijms20153775 PMC6696313 31382355

[70] Violi F, Oliva A, Cangemi R (2020). Nox2 activation in Covid-19. Redox Biol.

[71] Nguyen Dinh Cat A, Montezano AC, Burger D et al. Angiotensin II, NADPH oxidase, and redox signaling in the vasculature. Antioxid Redox Signal 2013; 19:1110–1120. 10.1089/ars.2012.4641 PMC3771549 22530599

[72] Somanath PR (2020). Is targeting Akt a viable option to treat advanced-stage COVID-19 patients?. Am J Physiol Lung Cell Mol Physiol.

[73] Artham S, Verma A, Alwhaibi A (2020). Delayed Akt suppression in the lipopolysaccharide-induced acute lung injury promotes resolution that is associated with enhanced effector regulatory T cells. Am J Physiol Lung Cell Mol Physiol.

[74] Berndt N, Yang H, Trinczek B (2010). The Akt activation inhibitor TCN-P inhibits Akt phosphorylation by binding to the PH domain of Akt and blocking its recruitment to the plasma membrane. Cell Death Differ.

[75] Xing Y, Lin NU, Maurer MA (2019). Phase II trial of AKT inhibitor MK-2206 in patients with advanced breast cancer who have tumors with PIK3CA or AKT mutations, and/or PTEN loss/PTEN mutation. Breast Cancer Res.

